# Supplementary material for: Mesenchymal stem cell-neural progenitors are enriched in cell signaling molecules implicated in their therapeutic effect in multiple sclerosis
Source: PLoS One. 2023 Aug 11;18(8):e0290069. doi: 10.1371/journal.pone.0290069 (PMC10420335; doi:10.1371/journal.pone.0290069)
Supplement: S1 Table — Gene ontology analysis was performed using GORilla. Target genes (“n”) were all 1,467 DEGs downregulated in MSC-NPs compared to MSCs, of which 1,234 genes were associated with a GO term. Background set of genes (“N”) consisted of all 24,196 genes detected by RNAseq, of which 17,705 genes were associated with a GO term. “B” is the total number of genes associated with each specific GO term, and “b” is the number of genes in the intersection. Enrichment factor = (b/n) / (B/N). Asterisks indicate pathways selected for manuscript. (PDF) [file pone.0290069.s001.pdf]

GO Term	GO Pathway Description	P-value	FDR q-value	-log10(FDR)	Enrichment Factor	N	B	n	b
GO:1903047	mitotic cell cycle process*	1.1E-33	1.7E-29	28.8	3.1	17705	608	1234	133
GO:0022402	cell cycle process	1.3E-29	9.8E-26	25.0	2.5	17705	957	1234	167
GO:0051301	cell division*	1.5E-22	7.4E-19	18.1	3.2	17705	386	1234	86
GO:0007010	cytoskeleton organization*	1.6E-20	6.1E-17	16.2	2.3	17705	860	1234	137
GO:0007059	chromosome segregation*	1.5E-18	4.6E-15	14.3	5.2	17705	108	1234	39
GO:0051276	chromosome organization	1.6E-17	4.2E-14	13.4	2.8	17705	400	1234	79
GO:0016043	cellular component organization	2.6E-17	5.8E-14	13.2	1.4	17705	5098	1234	488
GO:0071840	cellular component organization or biogenesis	1.6E-16	3.1E-13	12.5	1.4	17705	5144	1234	488
GO:0051726	regulation of cell cycle	5.3E-16	9.0E-13	12.0	1.9	17705	1143	1234	154
GO:0032502	developmental process	1.1E-14	1.7E-11	10.8	1.4	17705	4586	1234	437
GO:0098813	nuclear chromosome segregation	8.1E-14	1.1E-10	9.9	6.7	17705	47	1234	22
GO:0000819	sister chromatid segregation	1.3E-13	1.6E-10	9.8	7.8	17705	35	1234	19
GO:0000278	mitotic cell cycle	1.5E-13	1.8E-10	9.8	4.0	17705	133	1234	37
GO:0007088	regulation of mitotic nuclear division	1.5E-13	1.7E-10	9.8	3.7	17705	160	1234	41
GO:0000070	mitotic sister chromatid segregation	2.5E-13	2.5E-10	9.6	8.1	17705	32	1234	18
GO:0006996	organelle organization	2.5E-13	2.4E-10	9.6	1.5	17705	2359	1234	253
GO:0051983	regulation of chromosome segregation	3.0E-13	2.7E-10	9.6	4.3	17705	110	1234	33
GO:0007049	cell cycle	7.2E-13	6.2E-10	9.2	2.5	17705	428	1234	73
GO:0010564	regulation of cell cycle process	8.1E-13	6.6E-10	9.2	2.1	17705	743	1234	106
GO:0044770	cell cycle phase transition	9.9E-13	7.6E-10	9.1	2.9	17705	260	1234	53
GO:0010965	regulation of mitotic sister chromatid separation	1.6E-12	1.1E-09	8.9	5.5	17705	63	1234	24
GO:0030029	actin filament-based process	2.3E-12	1.6E-09	8.8	2.5	17705	393	1234	68
GO:0009987	cellular process	2.9E-12	2.0E-09	8.7	1.1	17705	13586	1234	1042
GO:0044772	mitotic cell cycle phase transition	3.6E-12	2.3E-09	8.6	2.9	17705	252	1234	51
GO:0051783	regulation of nuclear division	4.1E-12	2.5E-09	8.6	3.3	17705	183	1234	42
GO:0006260	DNA replication*	5.2E-12	3.0E-09	8.5	3.6	17705	148	1234	37
GO:0030036	actin cytoskeleton organization	5.9E-12	3.4E-09	8.5	2.6	17705	322	1234	59
GO:0030071	regulation of mitotic metaphase/anaphase transition	1.5E-11	8.0E-09	8.1	5.4	17705	58	1234	22
GO:1905818	regulation of chromosome separation	2.2E-11	1.2E-08	7.9	4.9	17705	70	1234	24
GO:0007346	regulation of mitotic cell cycle	3.1E-11	1.6E-08	7.8	2.1	17705	616	1234	89
GO:0000075	cell cycle checkpoint	4.6E-11	2.3E-08	7.6	3.7	17705	123	1234	32
GO:1902099	regulation of metaphase/anaphase transition of cell cycle	4.7E-11	2.3E-08	7.6	5.2	17705	61	1234	22
GO:0033047	regulation of mitotic sister chromatid segregation	8.3E-11	3.8E-08	7.4	4.7	17705	74	1234	24
GO:1902850	microtubule cytoskeleton organization involved in mitosis*	8.4E-11	3.8E-08	7.4	3.8	17705	112	1234	30
GO:0000226	microtubule cytoskeleton organization	9.3E-11	4.1E-08	7.4	2.3	17705	398	1234	65
GO:0033045	regulation of sister chromatid segregation	1.1E-10	4.6E-08	7.3	4.3	17705	87	1234	26

GO:0007051	spindle organization	1.6E-10	6.8E-08	7.2	3.4	17705	143	1234	34
GO:0044843	cell cycle G1/S phase transition	2.8E-10	1.1E-07	6.9	4.0	17705	97	1234	27
GO:0051383	kinetochore organization	4.0E-10	1.6E-07	6.8	9.1	17705	19	1234	12
GO:0007017	microtubule-based process	6.8E-10	2.6E-07	6.6	2.0	17705	643	1234	88
GO:0000082	G1/S transition of mitotic cell cycle	9.0E-10	3.4E-07	6.5	3.9	17705	95	1234	26
GO:0022610	biological adhesion	1.1E-09	4.0E-07	6.4	1.8	17705	891	1234	111
GO:0090068	positive regulation of cell cycle process	1.1E-09	4.1E-07	6.4	2.6	17705	276	1234	49
GO:0006928	movement of cell or subcellular component	1.2E-09	4.1E-07	6.4	1.6	17705	1348	1234	152
GO:0006334	nucleosome assembly	1.4E-09	4.9E-07	6.3	3.4	17705	132	1234	31
GO:0065004	protein-DNA complex assembly	1.5E-09	5.0E-07	6.3	2.8	17705	202	1234	40
GO:0007093	mitotic cell cycle checkpoint	2.2E-09	7.3E-07	6.1	3.9	17705	92	1234	25
GO:1903046	meiotic cell cycle process	2.3E-09	7.3E-07	6.1	3.1	17705	157	1234	34
GO:1901987	regulation of cell cycle phase transition	2.4E-09	7.5E-07	6.1	2.1	17705	449	1234	67
GO:0007155	cell adhesion	3.0E-09	9.2E-07	6.0	1.8	17705	885	1234	109
GO:0050793	regulation of developmental process	5.9E-09	1.8E-06	5.7	1.4	17705	2537	1234	248
GO:0051128	regulation of cellular component organization	6.0E-09	1.8E-06	5.8	1.4	17705	2475	1234	243
GO:1901990	regulation of mitotic cell cycle phase transition	9.1E-09	2.7E-06	5.6	2.1	17705	415	1234	62
GO:0007052	mitotic spindle organization	1.1E-08	3.0E-06	5.5	3.9	17705	85	1234	23
GO:0097435	supramolecular fiber organization	1.2E-08	3.2E-06	5.5	2.1	17705	437	1234	64
GO:0051302	regulation of cell division	1.2E-08	3.3E-06	5.5	3.0	17705	159	1234	33
GO:0006333	chromatin assembly or disassembly	1.4E-08	3.9E-06	5.4	3.6	17705	100	1234	25
GO:0071173	spindle assembly checkpoint	1.5E-08	4.1E-06	5.4	7.2	17705	24	1234	12
GO:0071174	mitotic spindle checkpoint	1.5E-08	4.0E-06	5.4	7.2	17705	24	1234	12
GO:0007094	mitotic spindle assembly checkpoint	1.5E-08	3.9E-06	5.4	7.2	17705	24	1234	12
GO:0006259	DNA metabolic process	1.6E-08	4.1E-06	5.4	1.8	17705	771	1234	96
GO:2000816	negative regulation of mitotic sister chromatid separation	2.0E-08	5.0E-06	5.3	6.4	17705	29	1234	13
GO:0045787	positive regulation of cell cycle	2.1E-08	5.0E-06	5.3	2.2	17705	366	1234	56
GO:0006270	DNA replication initiation	2.2E-08	5.3E-06	5.3	5.9	17705	34	1234	14
GO:0033046	negative regulation of sister chromatid segregation	2.2E-08	5.2E-06	5.3	5.9	17705	34	1234	14
GO:0031577	spindle checkpoint	2.8E-08	6.4E-06	5.2	6.9	17705	25	1234	12
GO:0000076	DNA replication checkpoint	3.2E-08	7.4E-06	5.1	8.4	17705	17	1234	10
GO:0048856	anatomical structure development	3.8E-08	8.5E-06	5.1	1.3	17705	3097	1234	288
GO:0051270	regulation of cellular component movement	4.0E-08	9.0E-06	5.0	1.7	17705	1029	1234	118
GO:0051094	positive regulation of developmental process	4.5E-08	9.9E-06	5.0	1.6	17705	1341	1234	145
GO:0030198	extracellular matrix organization*	4.5E-08	9.7E-06	5.0	2.2	17705	336	1234	52
GO:0045841	negative regulation of mitotic metaphase/anaphase transition	4.8E-08	1.0E-05	5.0	6.6	17705	26	1234	12
GO:0051985	negative regulation of chromosome segregation	5.3E-08	1.1E-05	5.0	5.6	17705	36	1234	14
GO:0031055	chromatin remodeling at centromere	5.4E-08	1.1E-05	5.0	4.9	17705	47	1234	16
GO:1905819	negative regulation of chromosome separation	5.4E-08	1.1E-05	5.0	6.0	17705	31	1234	13
GO:0048523	negative regulation of cellular process	6.0E-08	1.2E-05	4.9	1.3	17705	4607	1234	402

GO:0071824	protein-DNA complex subunit organization	6.2E-08	1.2E-05	4.9	2.5	17705	238	1234	41
GO:0045786	negative regulation of cell cycle	7.2E-08	1.4E-05	4.8	1.9	17705	550	1234	73
GO:0006940	regulation of smooth muscle contraction	7.7E-08	1.5E-05	4.8	4.5	17705	54	1234	17
GO:2000026	regulation of multicellular organismal development	7.8E-08	1.5E-05	4.8	1.4	17705	2038	1234	202
GO:0051716	cellular response to stimulus	8.4E-08	1.6E-05	4.8	1.4	17705	2778	1234	261
GO:0033048	negative regulation of mitotic sister chromatid segregation	8.6E-08	1.6E-05	4.8	5.8	17705	32	1234	13
GO:0042127	regulation of cell proliferation	9.6E-08	1.8E-05	4.7	1.5	17705	1535	1234	160
GO:0033043	regulation of organelle organization	9.9E-08	1.8E-05	4.7	1.6	17705	1253	1234	136
GO:0000281	mitotic cytokinesis	1.1E-07	1.9E-05	4.7	4.7	17705	49	1234	16
GO:0071103	DNA conformation change	1.1E-07	2.0E-05	4.7	2.8	17705	157	1234	31
GO:0048869	cellular developmental process	1.2E-07	2.2E-05	4.7	1.4	17705	2549	1234	242
GO:0031032	actomyosin structure organization	1.3E-07	2.2E-05	4.7	3.4	17705	96	1234	23
GO:1902100	negative regulation of metaphase/anaphase transition of cell cycle	1.3E-07	2.3E-05	4.6	6.2	17705	28	1234	12
GO:0036293	response to decreased oxygen levels	1.4E-07	2.3E-05	4.6	2.3	17705	272	1234	44
GO:0009653	anatomical structure morphogenesis	1.5E-07	2.6E-05	4.6	1.5	17705	1309	1234	140
GO:1904018	positive regulation of vasculature development*	1.7E-07	2.8E-05	4.5	2.5	17705	211	1234	37
GO:0032465	regulation of cytokinesis	2.0E-07	3.4E-05	4.5	3.6	17705	84	1234	21
GO:0048519	negative regulation of biological process	2.4E-07	4.0E-05	4.4	1.2	17705	5256	1234	446
GO:0001666	response to hypoxia*	2.7E-07	4.4E-05	4.4	2.3	17705	260	1234	42
GO:1901988	negative regulation of cell cycle phase transition	3.1E-07	5.0E-05	4.3	2.4	17705	243	1234	40
GO:0030335	positive regulation of cell migration	3.2E-07	5.1E-05	4.3	1.9	17705	529	1234	69
GO:2000145	regulation of cell motility	3.3E-07	5.2E-05	4.3	1.6	17705	956	1234	108
GO:0030334	regulation of cell migration	3.4E-07	5.3E-05	4.3	1.6	17705	900	1234	103
GO:0051239	regulation of multicellular organismal process	3.4E-07	5.3E-05	4.3	1.3	17705	3080	1234	281
GO:0070887	cellular response to chemical stimulus	3.5E-07	5.2E-05	4.3	1.5	17705	1602	1234	163
GO:0051272	positive regulation of cellular component movement	3.8E-07	5.7E-05	4.2	1.8	17705	563	1234	72
GO:1901991	negative regulation of mitotic cell cycle phase transition	3.9E-07	5.9E-05	4.2	2.4	17705	227	1234	38
GO:0034728	nucleosome organization	4.0E-07	5.9E-05	4.2	2.7	17705	166	1234	31
GO:0000725	recombinational repair	4.1E-07	5.9E-05	4.2	3.2	17705	102	1234	23
GO:0001525	angiogenesis*	4.1E-07	6.0E-05	4.2	2.3	17705	264	1234	42
GO:0030154	cell differentiation	4.6E-07	6.5E-05	4.2	1.4	17705	1767	1234	176
GO:0010948	negative regulation of cell cycle process	5.2E-07	7.4E-05	4.1	2.1	17705	333	1234	49
GO:2000147	positive regulation of cell motility	5.4E-07	7.6E-05	4.1	1.8	17705	547	1234	70
GO:0043062	extracellular structure organization	5.6E-07	7.8E-05	4.1	2.0	17705	383	1234	54
GO:0006937	regulation of muscle contraction	5.9E-07	8.2E-05	4.1	2.7	17705	152	1234	29
GO:0040012	regulation of locomotion	6.8E-07	9.4E-05	4.0	1.6	17705	1016	1234	112
GO:0034080	CENP-A containing nucleosome assembly	6.8E-07	9.3E-05	4.0	4.7	17705	43	1234	14
GO:0032879	regulation of localization	7.1E-07	9.6E-05	4.0	1.3	17705	2630	1234	244

GO:0040017	positive regulation of locomotion	7.5E-07	1.0E-04	4.0	1.8	17705	573	1234	72
GO:0061572	actin filament bundle organization	8.2E-07	1.1E-04	4.0	4.1	17705	56	1234	16
GO:0040011	locomotion	9.1E-07	1.2E-04	3.9	1.6	17705	999	1234	110
GO:0007015	actin filament organization	1.1E-06	1.5E-04	3.8	2.3	17705	255	1234	40
GO:0051382	kinetochore assembly	1.1E-06	1.4E-04	3.8	8.2	17705	14	1234	8
GO:0000724	double-strand break repair via homologous recombination	1.1E-06	1.4E-04	3.8	3.2	17705	100	1234	22
GO:0051493	regulation of cytoskeleton organization	1.3E-06	1.6E-04	3.8	1.8	17705	528	1234	67
GO:0048518	positive regulation of biological process	1.6E-06	2.0E-04	3.7	1.2	17705	5763	1234	477
GO:0070482	response to oxygen levels	1.6E-06	2.0E-04	3.7	2.1	17705	297	1234	44
GO:0018057	peptidyl-lysine oxidation	1.6E-06	2.0E-04	3.7	14.4	17705	5	1234	5
GO:0048870	cell motility	1.8E-06	2.3E-04	3.6	1.6	17705	910	1234	101
GO:0001932	regulation of protein phosphorylation	2.0E-06	2.4E-04	3.6	1.5	17705	1381	1234	141
GO:0006336	DNA replication-independent nucleosome assembly	2.1E-06	2.5E-04	3.6	4.1	17705	53	1234	15
GO:0045931	positive regulation of mitotic cell cycle	2.2E-06	2.6E-04	3.6	2.6	17705	153	1234	28
GO:0006302	double-strand break repair	2.2E-06	2.6E-04	3.6	2.4	17705	197	1234	33
GO:0022603	regulation of anatomical structure morphogenesis	2.4E-06	2.8E-04	3.6	1.5	17705	1089	1234	116
GO:0045930	negative regulation of mitotic cell cycle	2.6E-06	3.0E-04	3.5	2.1	17705	302	1234	44
GO:0034724	DNA replication-independent nucleosome organization	2.7E-06	3.1E-04	3.5	4.0	17705	54	1234	15
GO:0051240	positive regulation of multicellular organismal process	2.8E-06	3.2E-04	3.5	1.4	17705	1682	1234	165
GO:0000910	cytokinesis	2.8E-06	3.2E-04	3.5	3.4	17705	75	1234	18
GO:0061640	cytoskeleton-dependent cytokinesis	2.8E-06	3.2E-04	3.5	3.4	17705	75	1234	18
GO:0071241	cellular response to inorganic substance	3.1E-06	3.5E-04	3.5	2.3	17705	209	1234	34
GO:0030261	chromosome condensation	3.2E-06	3.6E-04	3.4	4.8	17705	36	1234	12
GO:0045839	negative regulation of mitotic nuclear division	3.3E-06	3.6E-04	3.4	4.4	17705	42	1234	13
GO:0009636	response to toxic substance	3.4E-06	3.8E-04	3.4	2.0	17705	345	1234	48
GO:0051303	establishment of chromosome localization	3.6E-06	4.0E-04	3.4	3.7	17705	62	1234	16
GO:0051784	negative regulation of nuclear division	4.0E-06	4.3E-04	3.4	4.1	17705	49	1234	14
GO:0051338	regulation of transferase activity	4.1E-06	4.4E-04	3.4	1.6	17705	973	1234	105
GO:0045132	meiotic chromosome segregation	4.2E-06	4.5E-04	3.3	7.2	17705	16	1234	8
GO:0050896	response to stimulus	4.4E-06	4.7E-04	3.3	1.2	17705	4744	1234	399
GO:0022414	reproductive process	4.6E-06	4.9E-04	3.3	1.5	17705	1235	1234	127
GO:0042325	regulation of phosphorylation	4.8E-06	5.0E-04	3.3	1.4	17705	1550	1234	153
GO:0090287	regulation of cellular response to growth factor stimulus	4.9E-06	5.1E-04	3.3	2.1	17705	280	1234	41
GO:0034330	cell junction organization	5.1E-06	5.3E-04	3.3	2.3	17705	223	1234	35
GO:0051310	metaphase plate congression	5.2E-06	5.3E-04	3.3	4.0	17705	50	1234	14
GO:0050794	regulation of cellular process	5.2E-06	5.4E-04	3.3	1.1	17705	9867	1234	762
GO:0045214	sarcomere organization	5.9E-06	6.0E-04	3.2	4.9	17705	32	1234	11
GO:0033044	regulation of chromosome organization	6.0E-06	6.1E-04	3.2	2.0	17705	342	1234	47

GO:0033554	cellular response to stress	6.2E-06	6.2E-04	3.2	1.4	17705	1594	1234	156
GO:0048522	positive regulation of cellular process	6.4E-06	6.4E-04	3.2	1.2	17705	5101	1234	424
GO:0010035	response to inorganic substance	6.5E-06	6.4E-04	3.2	1.8	17705	478	1234	60
GO:0050000	chromosome localization	7.0E-06	6.9E-04	3.2	3.5	17705	65	1234	16
GO:1904029	regulation of cyclin-dependent protein kinase activity	7.1E-06	6.9E-04	3.2	2.9	17705	103	1234	21
GO:0031570	DNA integrity checkpoint	7.5E-06	7.3E-04	3.1	3.2	17705	80	1234	18
GO:0048646	anatomical structure formation involved in morphogenesis	7.5E-06	7.3E-04	3.1	1.7	17705	655	1234	76
GO:0006974	cellular response to DNA damage stimulus	8.4E-06	8.1E-04	3.1	1.6	17705	747	1234	84
GO:0071248	cellular response to metal ion	8.8E-06	8.4E-04	3.1	2.4	17705	182	1234	30
GO:0051988	regulation of attachment of spindle microtubules to kinetochore	9.3E-06	8.8E-04	3.1	7.7	17705	13	1234	7
GO:0010634	positive regulation of epithelial cell migration	1.1E-05	1.1E-03	3.0	2.4	17705	166	1234	28
GO:0000083	regulation of transcription involved in G1/S transition of mitotic cell cycle	1.2E-05	1.1E-03	3.0	4.6	17705	34	1234	11
GO:0071456	cellular response to hypoxia	1.3E-05	1.2E-03	2.9	2.7	17705	115	1234	22
GO:0098609	cell-cell adhesion	1.3E-05	1.2E-03	2.9	1.8	17705	489	1234	60
GO:0000079	regulation of cyclin-dependent protein serine/threonine kinase activity	1.3E-05	1.2E-03	2.9	2.9	17705	99	1234	20
GO:0045766	positive regulation of angiogenesis	1.4E-05	1.3E-03	2.9	2.3	17705	186	1234	30
GO:0043486	histone exchange	1.4E-05	1.3E-03	2.9	3.7	17705	54	1234	14
GO:0016477	cell migration	1.4E-05	1.3E-03	2.9	1.6	17705	815	1234	89
GO:0006323	DNA packaging	1.5E-05	1.3E-03	2.9	4.2	17705	41	1234	12
GO:0035556	intracellular signal transduction	1.6E-05	1.4E-03	2.9	1.4	17705	1547	1234	150
GO:0050789	regulation of biological process	1.6E-05	1.4E-03	2.9	1.1	17705	10661	1234	812
GO:0008284	positive regulation of cell proliferation	1.6E-05	1.4E-03	2.8	1.6	17705	852	1234	92
GO:0042221	response to chemical	1.8E-05	1.6E-03	2.8	1.3	17705	2474	1234	223
GO:2001251	negative regulation of chromosome organization	1.9E-05	1.7E-03	2.8	2.6	17705	135	1234	24
GO:0032970	regulation of actin filament-based process	1.9E-05	1.7E-03	2.8	1.9	17705	378	1234	49
GO:0051093	negative regulation of developmental process	2.0E-05	1.7E-03	2.8	1.5	17705	938	1234	99
GO:0009719	response to endogenous stimulus	2.0E-05	1.7E-03	2.8	1.5	17705	974	1234	102
GO:0051347	positive regulation of transferase activity	2.2E-05	1.9E-03	2.7	1.6	17705	653	1234	74
GO:0070507	regulation of microtubule cytoskeleton organization	2.4E-05	2.0E-03	2.7	2.3	17705	182	1234	29
GO:0090257	regulation of muscle system process	2.4E-05	2.1E-03	2.7	2.2	17705	220	1234	33
GO:0030155	regulation of cell adhesion	2.6E-05	2.2E-03	2.7	1.6	17705	701	1234	78
GO:0051052	regulation of DNA metabolic process	2.6E-05	2.2E-03	2.7	1.8	17705	425	1234	53
GO:0090329	regulation of DNA-dependent DNA replication	2.7E-05	2.2E-03	2.7	3.7	17705	50	1234	13
GO:0019220	regulation of phosphate metabolic process	2.7E-05	2.2E-03	2.7	1.4	17705	1738	1234	164
GO:0051174	regulation of phosphorus metabolic process	2.7E-05	2.2E-03	2.7	1.4	17705	1738	1234	164
GO:0006281	DNA repair	3.0E-05	2.5E-03	2.6	1.7	17705	492	1234	59
GO:0045859	regulation of protein kinase activity	3.1E-05	2.5E-03	2.6	1.6	17705	762	1234	83

GO:0010038	response to metal ion	3.2E-05	2.6E-03	2.6	1.9	17705	333	1234	44
GO:0032886	regulation of microtubule-based process	3.4E-05	2.8E-03	2.6	2.2	17705	214	1234	32
GO:0043549	regulation of kinase activity	3.7E-05	3.0E-03	2.5	1.5	17705	871	1234	92
GO:0036294	cellular response to decreased oxygen levels	3.8E-05	3.0E-03	2.5	2.6	17705	123	1234	22
GO:0006275	regulation of DNA replication	3.8E-05	3.0E-03	2.5	2.7	17705	106	1234	20
GO:1901342	regulation of vasculature development	4.2E-05	3.3E-03	2.5	1.8	17705	379	1234	48
GO:0010595	positive regulation of endothelial cell migration	4.3E-05	3.4E-03	2.5	2.6	17705	124	1234	22
GO:0097237	cellular response to toxic substance	4.4E-05	3.4E-03	2.5	2.7	17705	107	1234	20
GO:0009628	response to abiotic stimulus	4.8E-05	3.7E-03	2.4	1.5	17705	1007	1234	103
GO:1901605	alpha-amino acid metabolic process	4.8E-05	3.7E-03	2.4	2.1	17705	208	1234	31
GO:0071459	protein localization to chromosome, centromeric region	4.9E-05	3.7E-03	2.4	5.5	17705	21	1234	8
GO:0051782	negative regulation of cell division	5.1E-05	3.9E-03	2.4	6.3	17705	16	1234	7
GO:0045765	regulation of angiogenesis	5.2E-05	4.0E-03	2.4	1.9	17705	340	1234	44
GO:0010810	regulation of cell-substrate adhesion	5.3E-05	4.0E-03	2.4	2.1	17705	209	1234	31
GO:0006310	DNA recombination	5.4E-05	4.1E-03	2.4	2.2	17705	190	1234	29
GO:0042327	positive regulation of phosphorylation	6.3E-05	4.7E-03	2.3	1.5	17705	1014	1234	103
GO:0031399	regulation of protein modification process	6.3E-05	4.7E-03	2.3	1.3	17705	1766	1234	164
GO:0034329	cell junction assembly	6.5E-05	4.8E-03	2.3	2.2	17705	173	1234	27
GO:1901701	cellular response to oxygen-containing compound	6.8E-05	5.0E-03	2.3	1.5	17705	791	1234	84
GO:0032501	multicellular organismal process	7.1E-05	5.2E-03	2.3	1.2	17705	3024	1234	261
GO:0000727	double-strand break repair via break-induced replication	7.3E-05	5.3E-03	2.3	7.2	17705	12	1234	6
GO:0051255	spindle midzone assembly	7.3E-05	5.3E-03	2.3	7.2	17705	12	1234	6
GO:1905065	positive regulation of vascular smooth muscle cell differentiation	7.3E-05	5.3E-03	2.3	7.2	17705	12	1234	6
GO:1901607	alpha-amino acid biosynthetic process	7.3E-05	5.2E-03	2.3	3.2	17705	62	1234	14
GO:0032467	positive regulation of cytokinesis	7.3E-05	5.2E-03	2.3	4.2	17705	34	1234	10
GO:0045143	homologous chromosome segregation	7.7E-05	5.5E-03	2.3	9.0	17705	8	1234	5
GO:0051256	mitotic spindle midzone assembly	7.7E-05	5.4E-03	2.3	9.0	17705	8	1234	5
GO:0061037	negative regulation of cartilage development	7.7E-05	5.5E-03	2.3	4.6	17705	28	1234	9
GO:0010562	positive regulation of phosphorus metabolic process	7.8E-05	5.5E-03	2.3	1.4	17705	1080	1234	108
GO:0045937	positive regulation of phosphate metabolic process	7.8E-05	5.5E-03	2.3	1.4	17705	1080	1234	108
GO:0008652	cellular amino acid biosynthetic process	7.9E-05	5.5E-03	2.3	2.9	17705	78	1234	16
GO:0042493	response to drug	7.9E-05	5.5E-03	2.3	1.6	17705	690	1234	75
GO:0065007	biological regulation	8.0E-05	5.6E-03	2.3	1.1	17705	11360	1234	853
GO:0009070	serine family amino acid biosynthetic process	8.2E-05	5.7E-03	2.2	5.9	17705	17	1234	7
GO:1901700	response to oxygen-containing compound	9.1E-05	6.2E-03	2.2	1.4	17705	1267	1234	123
GO:0044093	positive regulation of molecular function	9.2E-05	6.3E-03	2.2	1.3	17705	1678	1234	156
GO:0051261	protein depolymerization	9.6E-05	6.5E-03	2.2	4.1	17705	35	1234	10
GO:0003008	system process	9.8E-05	6.6E-03	2.2	1.4	17705	1220	1234	119

GO:1902749	regulation of cell cycle G2/M phase transition	9.9E-05	6.7E-03	2.2	2.1	17705	216	1234	31
GO:0071453	cellular response to oxygen levels	1.0E-04	6.7E-03	2.2	2.4	17705	140	1234	23
GO:0070527	platelet aggregation	1.0E-04	6.9E-03	2.2	3.8	17705	42	1234	11
GO:0032331	negative regulation of chondrocyte differentiation	1.0E-04	6.9E-03	2.2	5.0	17705	23	1234	8
GO:0008608	attachment of spindle microtubules to kinetochore	1.0E-04	6.9E-03	2.2	5.0	17705	23	1234	8
GO:0051130	positive regulation of cellular component organization	1.0E-04	6.8E-03	2.2	1.4	17705	1234	1234	120
GO:0043648	dicarboxylic acid metabolic process	1.1E-04	6.9E-03	2.2	2.8	17705	88	1234	17
GO:0009064	glutamine family amino acid metabolic process	1.1E-04	6.9E-03	2.2	3.1	17705	64	1234	14
GO:0051385	response to mineralocorticoid	1.1E-04	6.9E-03	2.2	4.5	17705	29	1234	9
GO:0071900	regulation of protein serine/threonine kinase activity	1.1E-04	6.9E-03	2.2	1.7	17705	492	1234	57
GO:0007165	signal transduction	1.1E-04	7.0E-03	2.2	1.2	17705	4140	1234	343
GO:0014910	regulation of smooth muscle cell migration	1.1E-04	7.0E-03	2.2	3.0	17705	72	1234	15
GO:1900158	negative regulation of bone mineralization involved in bone maturation	1.1E-04	7.1E-03	2.1	11.5	17705	5	1234	4
GO:0003018	vascular process in circulatory system	1.1E-04	7.1E-03	2.1	2.3	17705	141	1234	23
GO:0048513	animal organ development	1.1E-04	7.2E-03	2.1	1.4	17705	1212	1234	118
GO:0043085	positive regulation of catalytic activity	1.2E-04	7.3E-03	2.1	1.4	17705	1348	1234	129
GO:1903034	regulation of response to wounding	1.2E-04	7.4E-03	2.1	2.2	17705	179	1234	27
GO:0008150	biological_process	1.3E-04	8.0E-03	2.1	1.0	17705	16309	1234	1169
GO:0045005	DNA-dependent DNA replication maintenance of fidelity	1.3E-04	8.1E-03	2.1	3.7	17705	43	1234	11
GO:0010604	positive regulation of macromolecule metabolic process	1.3E-04	8.3E-03	2.1	1.2	17705	3198	1234	272
GO:0010389	regulation of G2/M transition of mitotic cell cycle	1.4E-04	8.5E-03	2.1	2.1	17705	200	1234	29
GO:0071495	cellular response to endogenous stimulus	1.4E-04	8.9E-03	2.1	1.5	17705	680	1234	73
GO:0034502	protein localization to chromosome	1.5E-04	9.2E-03	2.0	3.0	17705	66	1234	14
GO:0010639	negative regulation of organelle organization	1.5E-04	9.1E-03	2.0	1.7	17705	388	1234	47
GO:0001934	positive regulation of protein phosphorylation	1.5E-04	9.2E-03	2.0	1.5	17705	941	1234	95
GO:0071310	cellular response to organic substance	1.6E-04	9.4E-03	2.0	1.4	17705	1283	1234	123
GO:0051653	spindle localization	1.6E-04	9.4E-03	2.0	3.4	17705	51	1234	12
GO:0065009	regulation of molecular function	1.6E-04	9.7E-03	2.0	1.2	17705	2995	1234	256
GO:0048478	replication fork protection	1.6E-04	9.7E-03	2.0	8.0	17705	9	1234	5
GO:0000018	regulation of DNA recombination	1.7E-04	1.0E-02	2.0	2.6	17705	100	1234	18
GO:0051017	actin filament bundle assembly	1.9E-04	1.1E-02	2.0	3.3	17705	52	1234	12
GO:0050790	regulation of catalytic activity	1.9E-04	1.1E-02	1.9	1.3	17705	2372	1234	208
GO:0061041	regulation of wound healing	1.9E-04	1.1E-02	1.9	2.3	17705	146	1234	23
GO:0032392	DNA geometric change	2.0E-04	1.2E-02	1.9	2.5	17705	110	1234	19
GO:0010273	detoxification of copper ion	2.1E-04	1.2E-02	1.9	6.2	17705	14	1234	6
GO:0009893	positive regulation of metabolic process	2.1E-04	1.2E-02	1.9	1.2	17705	3487	1234	292
GO:0008283	cell proliferation	2.2E-04	1.3E-02	1.9	1.7	17705	405	1234	48
GO:0003013	circulatory system process	2.3E-04	1.3E-02	1.9	2.1	17705	186	1234	27

GO:0014070	response to organic cyclic compound	2.4E-04	1.4E-02	1.9	1.5	17705	680	1234	72
GO:0070301	cellular response to hydrogen peroxide	2.4E-04	1.4E-02	1.9	3.1	17705	61	1234	13
GO:1901989	positive regulation of cell cycle phase transition	2.4E-04	1.4E-02	1.9	2.6	17705	94	1234	17
GO:0043408	regulation of MAPK cascade	2.5E-04	1.4E-02	1.9	1.5	17705	692	1234	73
GO:0035690	cellular response to drug	2.5E-04	1.4E-02	1.9	1.8	17705	289	1234	37
GO:0051293	establishment of spindle localization	2.5E-04	1.4E-02	1.9	3.4	17705	46	1234	11
GO:0045595	regulation of cell differentiation	2.6E-04	1.5E-02	1.8	1.3	17705	1791	1234	162
GO:0090092	regulation of transmembrane receptor protein serine/threonine kinase signaling pathway	2.6E-04	1.5E-02	1.8	1.9	17705	238	1234	32
GO:0030324	lung development	2.8E-04	1.6E-02	1.8	2.8	17705	78	1234	15
GO:0051781	positive regulation of cell division	2.8E-04	1.6E-02	1.8	2.8	17705	78	1234	15
GO:0032508	DNA duplex unwinding	2.9E-04	1.6E-02	1.8	2.5	17705	104	1234	18
GO:0017015	regulation of transforming growth factor beta receptor signaling pathway	2.9E-04	1.6E-02	1.8	2.4	17705	122	1234	20
GO:1902807	negative regulation of cell cycle G1/S phase transition	2.9E-04	1.6E-02	1.8	2.4	17705	113	1234	19
GO:0060415	muscle tissue morphogenesis	2.9E-04	1.6E-02	1.8	3.0	17705	62	1234	13
GO:0048729	tissue morphogenesis	2.9E-04	1.6E-02	1.8	1.8	17705	334	1234	41
GO:0034332	adherens junction organization	3.0E-04	1.6E-02	1.8	2.6	17705	87	1234	16
GO:0060249	anatomical structure homeostasis	3.0E-04	1.7E-02	1.8	1.8	17705	292	1234	37
GO:0006563	L-serine metabolic process	3.1E-04	1.7E-02	1.8	7.2	17705	10	1234	5
GO:0033314	mitotic DNA replication checkpoint	3.1E-04	1.7E-02	1.8	7.2	17705	10	1234	5
GO:2000105	positive regulation of DNA-dependent DNA replication	3.1E-04	1.7E-02	1.8	7.2	17705	10	1234	5
GO:0009725	response to hormone	3.1E-04	1.7E-02	1.8	1.5	17705	616	1234	66
GO:1900157	regulation of bone mineralization involved in bone maturation	3.1E-04	1.7E-02	1.8	9.6	17705	6	1234	4
GO:0010837	regulation of keratinocyte proliferation	3.2E-04	1.7E-02	1.8	3.9	17705	33	1234	9
GO:0006936	muscle contraction	3.2E-04	1.7E-02	1.8	2.0	17705	200	1234	28
GO:0007080	mitotic metaphase plate congression	3.2E-04	1.7E-02	1.8	3.6	17705	40	1234	10
GO:0061687	detoxification of inorganic compound	3.3E-04	1.7E-02	1.8	5.7	17705	15	1234	6
GO:1903504	regulation of mitotic spindle checkpoint	3.3E-04	1.7E-02	1.8	5.7	17705	15	1234	6
GO:0090266	regulation of mitotic cell cycle spindle assembly checkpoint	3.3E-04	1.7E-02	1.8	5.7	17705	15	1234	6
GO:0090231	regulation of spindle checkpoint	3.3E-04	1.7E-02	1.8	5.7	17705	15	1234	6
GO:0003257	positive regulation of transcription from RNA polymerase II promoter involved in myocardial precursor cell differentiation	3.4E-04	1.8E-02	1.8	14.4	17705	3	1234	3
GO:0000086	G2/M transition of mitotic cell cycle	3.5E-04	1.8E-02	1.7	2.3	17705	133	1234	21
GO:0048545	response to steroid hormone	3.5E-04	1.8E-02	1.7	2.0	17705	201	1234	28
GO:0044057	regulation of system process	3.6E-04	1.8E-02	1.7	1.6	17705	538	1234	59
GO:0003012	muscle system process	3.6E-04	1.8E-02	1.7	1.9	17705	242	1234	32
GO:0022607	cellular component assembly	3.8E-04	1.9E-02	1.7	1.3	17705	2206	1234	193

GO:0045840	positive regulation of mitotic nuclear division	4.0E-04	2.0E-02	1.7	3.1	17705	56	1234	12
GO:1903844	regulation of cellular response to transforming growth factor beta stimulus	4.0E-04	2.0E-02	1.7	2.3	17705	125	1234	20
GO:0044839	cell cycle G2/M phase transition	4.2E-04	2.2E-02	1.7	2.2	17705	135	1234	21
GO:0006950	response to stress	4.3E-04	2.2E-02	1.7	1.2	17705	2856	1234	242
GO:0044774	mitotic DNA integrity checkpoint	4.5E-04	2.3E-02	1.6	3.2	17705	49	1234	11
GO:0033674	positive regulation of kinase activity	4.6E-04	2.3E-02	1.6	1.5	17705	578	1234	62
GO:0061045	negative regulation of wound healing	4.7E-04	2.3E-02	1.6	2.9	17705	65	1234	13
GO:0034109	homotypic cell-cell adhesion	4.7E-04	2.3E-02	1.6	3.0	17705	57	1234	12
GO:0032956	regulation of actin cytoskeleton organization	4.8E-04	2.4E-02	1.6	1.7	17705	342	1234	41
GO:0046677	response to antibiotic	4.8E-04	2.4E-02	1.6	1.8	17705	267	1234	34
GO:1904752	regulation of vascular associated smooth muscle cell migration	5.1E-04	2.5E-02	1.6	3.7	17705	35	1234	9
GO:0031297	replication fork processing	5.1E-04	2.5E-02	1.6	3.7	17705	35	1234	9
GO:1903011	negative regulation of bone development	5.3E-04	2.6E-02	1.6	6.5	17705	11	1234	5
GO:0090066	regulation of anatomical structure size	5.7E-04	2.8E-02	1.6	1.7	17705	378	1234	44
GO:0070848	response to growth factor	5.7E-04	2.8E-02	1.6	1.8	17705	291	1234	36
GO:0071236	cellular response to antibiotic	5.8E-04	2.8E-02	1.6	2.4	17705	110	1234	18
GO:2000134	negative regulation of G1/S transition of mitotic cell cycle	5.8E-04	2.8E-02	1.6	2.4	17705	110	1234	18
GO:0051247	positive regulation of protein metabolic process	6.0E-04	2.9E-02	1.5	1.3	17705	1593	1234	144
GO:0007062	sister chromatid cohesion	6.0E-04	2.9E-02	1.5	3.3	17705	43	1234	10
GO:0030193	regulation of blood coagulation	6.0E-04	2.9E-02	1.5	2.7	17705	75	1234	14
GO:0051173	positive regulation of nitrogen compound metabolic process	6.0E-04	2.9E-02	1.5	1.2	17705	3032	1234	254
GO:1902751	positive regulation of cell cycle G2/M phase transition	6.2E-04	3.0E-02	1.5	4.0	17705	29	1234	8
GO:0045740	positive regulation of DNA replication	6.4E-04	3.0E-02	1.5	3.6	17705	36	1234	9
GO:0050880	regulation of blood vessel size	6.4E-04	3.1E-02	1.5	2.3	17705	111	1234	18
GO:0097746	regulation of blood vessel diameter	6.4E-04	3.0E-02	1.5	2.3	17705	111	1234	18
GO:0035150	regulation of tube size	6.4E-04	3.0E-02	1.5	2.3	17705	111	1234	18
GO:0035296	regulation of tube diameter	6.4E-04	3.0E-02	1.5	2.3	17705	111	1234	18
GO:0010632	regulation of epithelial cell migration	6.5E-04	3.1E-02	1.5	1.8	17705	261	1234	33
GO:0070192	chromosome organization involved in meiotic cell cycle	6.5E-04	3.0E-02	1.5	2.9	17705	59	1234	12
GO:0031401	positive regulation of protein modification process	6.5E-04	3.0E-02	1.5	1.4	17705	1155	1234	109
GO:1900046	regulation of hemostasis	6.9E-04	3.2E-02	1.5	2.6	17705	76	1234	14
GO:0070601	centromeric sister chromatid cohesion	6.9E-04	3.2E-02	1.5	8.2	17705	7	1234	4
GO:1905278	positive regulation of epithelial tube formation	6.9E-04	3.2E-02	1.5	8.2	17705	7	1234	4
GO:0006520	cellular amino acid metabolic process	7.0E-04	3.2E-02	1.5	1.8	17705	294	1234	36
GO:0097305	response to alcohol	7.1E-04	3.3E-02	1.5	1.9	17705	210	1234	28
GO:0045597	positive regulation of cell differentiation	7.1E-04	3.2E-02	1.5	1.4	17705	960	1234	93
GO:0051246	regulation of protein metabolic process	7.1E-04	3.2E-02	1.5	1.2	17705	2668	1234	226

GO:1904753	negative regulation of vascular associated smooth muscle cell migration	7.2E-04	3.3E-02	1.5	5.1	17705	17	1234	6
GO:0043044	ATP-dependent chromatin remodeling	7.2E-04	3.3E-02	1.5	2.5	17705	85	1234	15
GO:0033993	response to lipid	7.3E-04	3.3E-02	1.5	1.5	17705	695	1234	71
GO:0051785	positive regulation of nuclear division	7.4E-04	3.3E-02	1.5	2.7	17705	68	1234	13
GO:0048660	regulation of smooth muscle cell proliferation	7.6E-04	3.4E-02	1.5	2.1	17705	141	1234	21
GO:0010033	response to organic substance	7.8E-04	3.5E-02	1.5	1.3	17705	1990	1234	174
GO:0008285	negative regulation of cell proliferation	7.9E-04	3.5E-02	1.5	1.5	17705	685	1234	70
GO:0016572	histone phosphorylation	8.0E-04	3.6E-02	1.4	3.8	17705	30	1234	8
GO:0051129	negative regulation of cellular component organization	8.0E-04	3.6E-02	1.4	1.5	17705	733	1234	74
GO:0031424	keratinization	8.3E-04	3.7E-02	1.4	2.4	17705	95	1234	16
GO:1904705	regulation of vascular smooth muscle cell proliferation	8.5E-04	3.8E-02	1.4	2.7	17705	69	1234	13
GO:0072422	signal transduction involved in DNA damage checkpoint	8.5E-04	3.7E-02	1.4	2.7	17705	69	1234	13
GO:0072401	signal transduction involved in DNA integrity checkpoint	8.5E-04	3.7E-02	1.4	2.7	17705	69	1234	13
GO:0018158	protein oxidation	8.5E-04	3.7E-02	1.4	6.0	17705	12	1234	5
GO:0030042	actin filament depolymerization	8.5E-04	3.7E-02	1.4	6.0	17705	12	1234	5
GO:0007077	mitotic nuclear envelope disassembly	8.5E-04	3.7E-02	1.4	6.0	17705	12	1234	5
GO:0060707	trophoblast giant cell differentiation	8.5E-04	3.7E-02	1.4	6.0	17705	12	1234	5
GO:0051315	attachment of mitotic spindle microtubules to kinetochore	8.5E-04	3.7E-02	1.4	6.0	17705	12	1234	5
GO:0051764	actin crosslink formation	8.5E-04	3.7E-02	1.4	6.0	17705	12	1234	5
GO:0060347	heart trabecula formation	8.5E-04	3.7E-02	1.4	6.0	17705	12	1234	5
GO:0065008	regulation of biological quality	8.6E-04	3.7E-02	1.4	1.2	17705	3561	1234	292
GO:1902806	regulation of cell cycle G1/S phase transition	8.7E-04	3.7E-02	1.4	2.0	17705	172	1234	24
GO:0030199	collagen fibril organization	8.8E-04	3.8E-02	1.4	3.2	17705	45	1234	10
GO:0016051	carbohydrate biosynthetic process	8.9E-04	3.8E-02	1.4	2.3	17705	114	1234	18
GO:0010043	response to zinc ion	9.1E-04	3.9E-02	1.4	3.0	17705	53	1234	11
GO:0006325	chromatin organization	9.3E-04	3.9E-02	1.4	1.5	17705	689	1234	70
GO:0032270	positive regulation of cellular protein metabolic process	9.4E-04	4.0E-02	1.4	1.3	17705	1521	1234	137
GO:0051147	regulation of muscle cell differentiation	9.5E-04	4.0E-02	1.4	2.0	17705	163	1234	23
GO:0090288	negative regulation of cellular response to growth factor stimulus	9.5E-04	4.0E-02	1.4	2.0	17705	163	1234	23
GO:0045987	positive regulation of smooth muscle contraction	9.5E-04	4.0E-02	1.4	4.2	17705	24	1234	7
GO:0009065	glutamine family amino acid catabolic process	9.5E-04	4.0E-02	1.4	4.2	17705	24	1234	7
