# Supplementary material for: Mesenchymal stem cell-neural progenitors are enriched in cell signaling molecules implicated in their therapeutic effect in multiple sclerosis
Source: PLoS One. 2023 Aug 11;18(8):e0290069. doi: 10.1371/journal.pone.0290069 (PMC10420335; doi:10.1371/journal.pone.0290069)
Supplement: S2 Table — (PDF) [file pone.0290069.s002.pdf]

Gene names – mitotic cell cycle process (GO:1903047)			
AJUBA	CENPK	KIF18B	PRIM2
ANLN	CEP55	KIF20A	PSRC1
AURKA	CEP72	KIF22	RACGAP1
AURKB	CIT	KIF23	RAD51
BLM	CKAP2	KIF2C	RAN
BORA	CKS2	KIF4A	REEP4
BRCA1	CLSPN	KNSTRN	RHOB
BRCA2	DBF4	KNTC1	RRM2
BUB1	DHFR	MAD2L1	SAPCD2
BUB1B	DLGAP5	MCM10	SFN
CCNA2	DNA2	MCM2	SGOL1
CCNB1	E2F1	MCM4	SGOL2
CCNB2	E2F7	MCM5	SKP2
CCNE1	E2F8	MCM8	SMC4
CCNE2	ECT2	MELK	SPAG5
CCNF	EIF4EBP1	MYBL2	SPC25
CDC20	EME1	MYC	SPDL1
CDC25A	ESPL1	NCAPG	STIL
CDC25C	FANCD2	NCAPG2	STK33
CDC45	FBXL22	NCAPH	STMN1
CDC6	FLNA	NDC80	TACC3
CDCA5	FOXM1	NEK2	TICRR
CDCA8	GINS1	NUF2	TIPIN
CDK1	GINS3	NUSAP1	TPD52L1
CDK15	GSG2	ORC1	TPX2
CDKN2C	GTSE1	ORC6	TRIP13
CDKN3	HUS1B	PKMYT1	TTK
CDT1	INCENP	PLK1	TUBB4B
CENPA	INHBA	PLK2	TYMS
CENPE	IQGAP3	PLK4	UBE2C
CENPF	KIF11	POLE2	UBE2S
CENPH	KIF14	PRC1	VRK1
CENPJ	KIF18A	PRIM1	WDR62
			ZWINT
