# Supplementary material for: Mesenchymal stem cell-neural progenitors are enriched in cell signaling molecules implicated in their therapeutic effect in multiple sclerosis
Source: PLoS One. 2023 Aug 11;18(8):e0290069. doi: 10.1371/journal.pone.0290069 (PMC10420335; doi:10.1371/journal.pone.0290069)
Supplement: S3 Table — Gene ontology analysis was performed using GORilla. Target genes (“n”) were all 2,156 DEGs upregulated in MSC-NPs compared to MSCs, of which 1,799 genes were associated with a GO term. Background set of genes (“N”) consisted of all 24,196 genes detected by RNAseq, of which 17,707 genes were associated with a GO term. “B” is the total number of genes associated with each specific GO term, and “b” is the number of genes in the intersection. Enrichment factor = (b/n) / (B/N). Asterisks indicate pathways selected for manuscript. (PDF) [file pone.0290069.s003.pdf]

GO Term	GO pathway description	P-value	FDR q-value	-log10(FDR)	Enrichment Factor	N	B	n	b
GO:0007155	cell adhesion	3.3E-30	5.0E-26	25.3	2.3	17707	885	1799	203
GO:0022610	biological adhesion	8.6E-30	6.6E-26	25.2	2.2	17707	891	1799	203
GO:0051239	regulation of multicellular organismal process	2.2E-28	1.1E-24	24.0	1.6	17707	3080	1799	490
GO:0098742	cell-cell adhesion via plasma-membrane adhesion molecules	5.8E-27	2.2E-23	22.6	3.5	17707	245	1799	87
GO:0098609	cell-cell adhesion*	1.3E-24	3.8E-21	20.4	2.6	17707	489	1799	128
GO:0007156	homophilic cell adhesion via plasma membrane adhesion molecules	5.1E-24	1.3E-20	19.9	4.0	17707	161	1799	65
GO:0050793	regulation of developmental process	1.0E-23	2.3E-20	19.6	1.6	17707	2537	1799	407
GO:0023052	signaling*	4.0E-21	7.6E-18	17.1	2.3	17707	608	1799	140
GO:2000145	regulation of cell motility	1.7E-20	2.9E-17	16.5	2.0	17707	956	1799	190
GO:0007267	cell-cell signaling*	4.1E-20	6.2E-17	16.2	2.3	17707	533	1799	126
GO:0051270	regulation of cellular component movement	5.0E-20	7.0E-17	16.2	1.9	17707	1029	1799	199
GO:2000026	regulation of multicellular organismal development	7.7E-20	9.9E-17	16.0	1.6	17707	2038	1799	331
GO:0040012	regulation of locomotion	1.3E-19	1.4E-16	15.8	1.9	17707	1016	1799	196
GO:0045595	regulation of cell differentiation	1.3E-19	1.5E-16	15.8	1.6	17707	1791	1799	299
GO:0032879	regulation of localization	1.2E-18	1.2E-15	14.9	1.5	17707	2630	1799	399
GO:0007166	cell surface receptor signaling pathway	2.9E-18	2.8E-15	14.6	1.6	17707	2069	1799	329
GO:0007154	cell communication*	3.2E-18	2.9E-15	14.5	2.0	17707	789	1799	160
GO:0007165	signal transduction	4.6E-18	3.9E-15	14.4	1.4	17707	4142	1799	572
GO:0030334	regulation of cell migration	1.5E-17	1.2E-14	13.9	1.9	17707	900	1799	174
GO:0048583	regulation of response to stimulus	8.6E-17	6.6E-14	13.2	1.4	17707	3984	1799	548
GO:0032101	regulation of response to external stimulus	1.9E-16	1.4E-13	12.9	1.9	17707	830	1799	161
GO:0051094	positive regulation of developmental process	3.8E-16	2.6E-13	12.6	1.7	17707	1341	1799	229
GO:0043062	extracellular structure organization	6.4E-16	4.3E-13	12.4	2.4	17707	383	1799	93
GO:0051960	regulation of nervous system development*	7.6E-16	4.9E-13	12.3	1.9	17707	871	1799	165
GO:0045597	positive regulation of cell differentiation	2.0E-15	1.2E-12	11.9	1.8	17707	960	1799	176
GO:0030198	extracellular matrix organization*	2.8E-15	1.7E-12	11.8	2.5	17707	336	1799	84
GO:0006954	inflammatory response*	2.9E-15	1.7E-12	11.8	2.3	17707	417	1799	97
GO:0006952	defense response	4.0E-15	2.2E-12	11.7	1.8	17707	1004	1799	181
GO:0051241	negative regulation of multicellular organismal process	4.6E-14	2.4E-11	10.6	1.7	17707	1197	1799	203
GO:0002682	regulation of immune system process	9.2E-14	4.7E-11	10.3	1.6	17707	1479	1799	238
GO:0006935	chemotaxis*	1.3E-13	6.3E-11	10.2	2.4	17707	302	1799	75
GO:0002685	regulation of leukocyte migration*	1.6E-13	7.6E-11	10.1	2.8	17707	198	1799	57
GO:0042330	taxis	1.8E-13	8.5E-11	10.1	2.4	17707	304	1799	75
GO:0023051	regulation of signaling	3.7E-13	1.7E-10	9.8	1.3	17707	3371	1799	460
GO:0048856	anatomical structure development	5.3E-13	2.3E-10	9.6	1.4	17707	3098	1799	428
GO:0060284	regulation of cell development	5.2E-13	2.3E-10	9.6	1.8	17707	906	1799	161
GO:0050767	regulation of neurogenesis*	7.7E-13	3.2E-10	9.5	1.8	17707	777	1799	143

GO:0051240	positive regulation of multicellular organismal process	1.1E-12	4.3E-10	9.4	1.5	17707	1682	1799	259
GO:0010646	regulation of cell communication	1.1E-12	4.4E-10	9.4	1.3	17707	3332	1799	453
GO:0048584	positive regulation of response to stimulus	1.6E-12	6.0E-10	9.2	1.4	17707	2176	1799	318
GO:0051271	negative regulation of cellular component movement	2.1E-12	7.8E-10	9.1	2.3	17707	337	1799	78
GO:0032502	developmental process	2.4E-12	8.7E-10	9.1	1.3	17707	4587	1799	591
GO:0040011	locomotion	4.9E-12	1.8E-09	8.8	1.7	17707	999	1799	170
GO:0030336	negative regulation of cell migration	1.4E-11	5.0E-09	8.3	2.3	17707	292	1799	69
GO:2000146	negative regulation of cell motility	1.5E-11	5.3E-09	8.3	2.3	17707	305	1799	71
GO:0050920	regulation of chemotaxis	2.2E-11	7.3E-09	8.1	2.6	17707	215	1799	56
GO:0042127	regulation of cell proliferation	2.7E-11	9.0E-09	8.0	1.5	17707	1536	1799	235
GO:0040013	negative regulation of locomotion	3.5E-11	1.1E-08	7.9	2.2	17707	349	1799	77
GO:0045664	regulation of neuron differentiation*	3.8E-11	1.2E-08	7.9	1.8	17707	626	1799	117
GO:0065008	regulation of biological quality	4.5E-11	1.4E-08	7.9	1.3	17707	3563	1799	470
GO:0032501	multicellular organismal process	4.8E-11	1.4E-08	7.8	1.3	17707	3024	1799	409
GO:0048731	system development	9.4E-11	2.8E-08	7.6	1.8	17707	686	1799	124
GO:0042221	response to chemical	1.1E-10	3.1E-08	7.5	1.4	17707	2475	1799	344
GO:0009605	response to external stimulus	1.1E-10	3.2E-08	7.5	1.5	17707	1379	1799	213
GO:0010033	response to organic substance	1.2E-10	3.3E-08	7.5	1.4	17707	1991	1799	287
GO:0099537	trans-synaptic signaling	1.9E-10	5.3E-08	7.3	2.3	17707	289	1799	66
GO:0099536	synaptic signaling*	3.1E-10	8.1E-08	7.1	2.2	17707	292	1799	66
GO:0048585	negative regulation of response to stimulus	3.0E-10	8.1E-08	7.1	1.5	17707	1607	1799	239
GO:2000147	positive regulation of cell motility	3.8E-10	9.8E-08	7.0	1.9	17707	547	1799	103
GO:0051962	positive regulation of nervous system development	4.1E-10	1.1E-07	7.0	1.9	17707	505	1799	97
GO:0032103	positive regulation of response to external stimulus	6.0E-10	1.5E-07	6.8	2.2	17707	303	1799	67
GO:0040017	positive regulation of locomotion	5.9E-10	1.5E-07	6.8	1.8	17707	573	1799	106
GO:0051272	positive regulation of cellular component movement	9.4E-10	2.3E-07	6.6	1.8	17707	563	1799	104
GO:0045596	negative regulation of cell differentiation	1.2E-09	2.8E-07	6.6	1.7	17707	683	1799	120
GO:0010721	negative regulation of cell development	1.4E-09	3.2E-07	6.5	2.2	17707	302	1799	66
GO:0051093	negative regulation of developmental process	1.4E-09	3.4E-07	6.5	1.6	17707	938	1799	153
GO:0002684	positive regulation of immune system process	2.5E-09	5.7E-07	6.2	1.6	17707	961	1799	155
GO:0044057	regulation of system process	3.0E-09	6.7E-07	6.2	1.8	17707	538	1799	99
GO:0050896	response to stimulus	3.0E-09	6.7E-07	6.2	1.2	17707	4745	1799	588
GO:0023056	positive regulation of signaling	3.4E-09	7.5E-07	6.1	1.4	17707	1716	1799	247
GO:0010647	positive regulation of cell communication	3.5E-09	7.6E-07	6.1	1.4	17707	1708	1799	246
GO:0030278	regulation of ossification*	4.4E-09	9.4E-07	6.0	2.4	17707	195	1799	48
GO:0002376	immune system process	4.8E-09	1.0E-06	6.0	1.4	17707	1985	1799	278
GO:0009966	regulation of signal transduction	5.4E-09	1.1E-06	6.0	1.3	17707	2981	1799	392
GO:0002688	regulation of leukocyte chemotaxis*	5.8E-09	1.2E-06	5.9	2.9	17707	115	1799	34

GO:0007268	chemical synaptic transmission	6.0E-09	1.2E-06	5.9	2.2	17707	273	1799	60
GO:0052547	regulation of peptidase activity	6.1E-09	1.2E-06	5.9	1.9	17707	416	1799	81
GO:0098916	anterograde trans-synaptic signaling	6.0E-09	1.2E-06	5.9	2.2	17707	273	1799	60
GO:0001501	skeletal system development	7.2E-09	1.4E-06	5.9	2.7	17707	144	1799	39
GO:0050921	positive regulation of chemotaxis	9.8E-09	1.9E-06	5.7	2.7	17707	134	1799	37
GO:0048518	positive regulation of biological process	1.0E-08	2.0E-06	5.7	1.2	17707	5764	1799	693
GO:0030335	positive regulation of cell migration	1.1E-08	2.0E-06	5.7	1.8	17707	529	1799	96
GO:0010975	regulation of neuron projection development	1.3E-08	2.3E-06	5.6	1.8	17707	487	1799	90
GO:0010817	regulation of hormone levels	1.4E-08	2.5E-06	5.6	1.8	17707	495	1799	91
GO:0050804	modulation of chemical synaptic transmission	1.4E-08	2.5E-06	5.6	1.9	17707	409	1799	79
GO:0099177	regulation of trans-synaptic signaling	1.5E-08	2.7E-06	5.6	1.9	17707	410	1799	79
GO:0050807	regulation of synapse organization	1.7E-08	3.0E-06	5.5	2.3	17707	209	1799	49
GO:0008285	negative regulation of cell proliferation	1.9E-08	3.3E-06	5.5	1.7	17707	685	1799	116
GO:0048870	cell motility	1.9E-08	3.3E-06	5.5	1.6	17707	910	1799	145
GO:0002687	positive regulation of leukocyte migration	2.2E-08	3.8E-06	5.4	2.7	17707	132	1799	36
GO:0022603	regulation of anatomical structure morphogenesis	2.4E-08	4.0E-06	5.4	1.5	17707	1089	1799	167
GO:0016477	cell migration	3.2E-08	5.3E-06	5.3	1.6	17707	815	1799	132
GO:0001934	positive regulation of protein phosphorylation	3.4E-08	5.5E-06	5.3	1.6	17707	942	1799	148
GO:0051049	regulation of transport	3.5E-08	5.7E-06	5.2	1.4	17707	1650	1799	234
GO:0051046	regulation of secretion	3.9E-08	6.3E-06	5.2	1.7	17707	580	1799	101
GO:0050727	regulation of inflammatory response	5.2E-08	8.3E-06	5.1	1.9	17707	407	1799	77
GO:0032102	negative regulation of response to external stimulus	5.5E-08	8.6E-06	5.1	2.0	17707	337	1799	67
GO:0070887	cellular response to chemical stimulus	6.5E-08	1.0E-05	5.0	1.4	17707	1603	1799	227
GO:0050808	synapse organization	6.4E-08	1.0E-05	5.0	2.3	17707	192	1799	45
GO:0010648	negative regulation of cell communication	7.8E-08	1.2E-05	4.9	1.4	17707	1330	1799	194
GO:0006022	aminoglycan metabolic process	7.8E-08	1.2E-05	4.9	2.5	17707	156	1799	39
GO:0050768	negative regulation of neurogenesis	7.7E-08	1.2E-05	4.9	2.1	17707	258	1799	55
GO:0048245	eosinophil chemotaxis	8.0E-08	1.2E-05	4.9	6.4	17707	17	1799	11
GO:0050769	positive regulation of neurogenesis	8.7E-08	1.3E-05	4.9	1.8	17707	448	1799	82
GO:0023057	negative regulation of signaling	9.2E-08	1.4E-05	4.9	1.4	17707	1333	1799	194
GO:0071310	cellular response to organic substance	9.6E-08	1.4E-05	4.9	1.4	17707	1284	1799	188
GO:0060326	cell chemotaxis	9.8E-08	1.4E-05	4.9	2.3	17707	182	1799	43
GO:0042327	positive regulation of phosphorylation	1.0E-07	1.5E-05	4.8	1.5	17707	1015	1799	155
GO:0009653	anatomical structure morphogenesis	1.0E-07	1.5E-05	4.8	1.4	17707	1310	1799	191
GO:0030595	leukocyte chemotaxis	1.1E-07	1.5E-05	4.8	2.6	17707	128	1799	34
GO:0030155	regulation of cell adhesion	1.3E-07	1.8E-05	4.8	1.6	17707	701	1799	115
GO:0051963	regulation of synapse assembly	1.3E-07	1.8E-05	4.7	2.9	17707	95	1799	28
GO:0052548	regulation of endopeptidase activity	1.5E-07	2.0E-05	4.7	1.9	17707	388	1799	73
GO:0010466	negative regulation of peptidase activity	1.5E-07	2.0E-05	4.7	2.2	17707	223	1799	49
GO:0051961	negative regulation of nervous system development	1.6E-07	2.1E-05	4.7	2.0	17707	277	1799	57

GO:0006955	immune response	1.7E-07	2.2E-05	4.7	1.5	17707	862	1799	135
GO:0043269	regulation of ion transport	1.8E-07	2.3E-05	4.6	1.6	17707	659	1799	109
GO:1903530	regulation of secretion by cell	1.8E-07	2.4E-05	4.6	1.7	17707	530	1799	92
GO:0072677	eosinophil migration	1.9E-07	2.4E-05	4.6	6.0	17707	18	1799	11
GO:0009967	positive regulation of signal transduction	1.9E-07	2.4E-05	4.6	1.4	17707	1565	1799	220
GO:0045937	positive regulation of phosphate metabolic process	3.0E-07	3.7E-05	4.4	1.5	17707	1081	1799	161
GO:0009607	response to biotic stimulus	3.0E-07	3.7E-05	4.4	1.6	17707	791	1799	125
GO:0010562	positive regulation of phosphorus metabolic process	3.0E-07	3.7E-05	4.4	1.5	17707	1081	1799	161
GO:0019932	second-messenger-mediated signaling	3.1E-07	3.8E-05	4.4	2.0	17707	303	1799	60
GO:0050900	leukocyte migration	3.4E-07	4.2E-05	4.4	2.0	17707	283	1799	57
GO:0050865	regulation of cell activation	3.6E-07	4.3E-05	4.4	1.7	17707	530	1799	91
GO:0002683	negative regulation of immune system process	3.8E-07	4.6E-05	4.3	1.8	17707	456	1799	81
GO:0043207	response to external biotic stimulus	4.1E-07	4.9E-05	4.3	1.6	17707	764	1799	121
GO:1902533	positive regulation of intracellular signal transduction	4.2E-07	5.0E-05	4.3	1.5	17707	1038	1799	155
GO:0007416	synapse assembly	4.3E-07	5.1E-05	4.3	3.0	17707	78	1799	24
GO:0065009	regulation of molecular function	4.4E-07	5.2E-05	4.3	1.3	17707	2997	1799	381
GO:0050776	regulation of immune response	4.4E-07	5.2E-05	4.3	1.5	17707	933	1799	142
GO:0016339	calcium-dependent cell-cell adhesion via plasma membrane cell adhesion molecules	4.7E-07	5.4E-05	4.3	4.0	17707	39	1799	16
GO:0014070	response to organic cyclic compound	4.9E-07	5.6E-05	4.3	1.6	17707	680	1799	110
GO:0010951	negative regulation of endopeptidase activity	5.1E-07	5.8E-05	4.2	2.1	17707	212	1799	46
GO:0043410	positive regulation of MAPK cascade	5.3E-07	6.0E-05	4.2	1.7	17707	512	1799	88
GO:1901700	response to oxygen-containing compound	6.2E-07	6.9E-05	4.2	1.4	17707	1268	1799	182
GO:0070372	regulation of ERK1 and ERK2 cascade	6.9E-07	7.6E-05	4.1	2.0	17707	282	1799	56
GO:0009968	negative regulation of signal transduction	6.9E-07	7.7E-05	4.1	1.4	17707	1245	1799	179
GO:0009719	response to endogenous stimulus	7.5E-07	8.2E-05	4.1	1.5	17707	974	1799	146
GO:0031347	regulation of defense response	7.7E-07	8.4E-05	4.1	1.5	17707	805	1799	125
GO:0007186	G protein-coupled receptor signaling pathway	7.7E-07	8.4E-05	4.1	1.5	17707	829	1799	128
GO:0045665	negative regulation of neuron differentiation	8.0E-07	8.6E-05	4.1	2.1	17707	202	1799	44
GO:0010720	positive regulation of cell development	8.4E-07	8.9E-05	4.0	1.7	17707	525	1799	89
GO:0048869	cellular developmental process	8.4E-07	8.9E-05	4.0	1.3	17707	2549	1799	329
GO:0051047	positive regulation of secretion	8.7E-07	9.1E-05	4.0	2.0	17707	277	1799	55
GO:0009887	animal organ morphogenesis	1.0E-06	1.1E-04	4.0	1.7	17707	482	1799	83
GO:0002690	positive regulation of leukocyte chemotaxis	1.1E-06	1.1E-04	4.0	2.8	17707	87	1799	25
GO:0048522	positive regulation of cellular process	1.2E-06	1.2E-04	3.9	1.2	17707	5102	1799	606
GO:0002686	negative regulation of leukocyte migration	1.2E-06	1.2E-04	3.9	3.6	17707	46	1799	17
GO:2000738	positive regulation of stem cell differentiation	1.6E-06	1.6E-04	3.8	5.2	17707	21	1799	11
GO:0002694	regulation of leukocyte activation	1.6E-06	1.6E-04	3.8	1.7	17707	495	1799	84
GO:0009612	response to mechanical stimulus	1.8E-06	1.8E-04	3.7	2.2	17707	188	1799	41
GO:0001932	regulation of protein phosphorylation	1.8E-06	1.8E-04	3.7	1.4	17707	1382	1799	193

GO:0070098	chemokine-mediated signaling pathway	1.9E-06	1.9E-04	3.7	3.0	17707	73	1799	22
GO:0065007	biological regulation	2.0E-06	1.9E-04	3.7	1.1	17707	11362	1799	1243
GO:0048878	chemical homeostasis	2.0E-06	2.0E-04	3.7	1.4	17707	1032	1799	151
GO:1903522	regulation of blood circulation	2.2E-06	2.1E-04	3.7	1.9	17707	264	1799	52
GO:0043408	regulation of MAPK cascade	2.2E-06	2.1E-04	3.7	1.6	17707	693	1799	109
GO:0006869	lipid transport	2.2E-06	2.1E-04	3.7	1.9	17707	264	1799	52
GO:0031344	regulation of cell projection organization	2.3E-06	2.2E-04	3.7	1.6	17707	670	1799	106
GO:0048513	animal organ development	2.8E-06	2.6E-04	3.6	1.4	17707	1213	1799	172
GO:0031644	regulation of neurological system process	2.8E-06	2.6E-04	3.6	2.4	17707	127	1799	31
GO:0051346	negative regulation of hydrolase activity	3.1E-06	2.9E-04	3.5	1.7	17707	412	1799	72
GO:2000501	regulation of natural killer cell chemotaxis	3.3E-06	3.1E-04	3.5	7.7	17707	9	1799	7
GO:0014059	regulation of dopamine secretion	4.2E-06	3.9E-04	3.4	4.1	17707	31	1799	13
GO:0033993	response to lipid	4.3E-06	3.9E-04	3.4	1.5	17707	695	1799	108
GO:0048545	response to steroid hormone	4.4E-06	4.0E-04	3.4	2.1	17707	201	1799	42
GO:0018146	keratan sulfate biosynthetic process	4.7E-06	4.3E-04	3.4	4.4	17707	27	1799	12
GO:0070374	positive regulation of ERK1 and ERK2 cascade	4.8E-06	4.4E-04	3.4	2.1	17707	195	1799	41
GO:0042592	homeostatic process	6.1E-06	5.5E-04	3.3	1.4	17707	1425	1799	195
GO:0019221	cytokine-mediated signaling pathway	6.6E-06	5.9E-04	3.2	1.6	17707	630	1799	99
GO:0046903	secretion	8.4E-06	7.5E-04	3.1	1.4	17707	967	1799	140
GO:0003013	circulatory system process	8.7E-06	7.7E-04	3.1	2.1	17707	186	1799	39
GO:0050790	regulation of catalytic activity	8.8E-06	7.7E-04	3.1	1.3	17707	2373	1799	302
GO:0097164	ammonium ion metabolic process	9.0E-06	7.8E-04	3.1	2.0	17707	193	1799	40
GO:0042445	hormone metabolic process	9.0E-06	7.9E-04	3.1	2.0	17707	193	1799	40
GO:0120035	regulation of plasma membrane bounded cell projection organization	1.1E-05	9.2E-04	3.0	1.5	17707	661	1799	102
GO:0045666	positive regulation of neuron differentiation	1.1E-05	9.7E-04	3.0	1.7	17707	352	1799	62
GO:0032570	response to progesterone	1.2E-05	9.8E-04	3.0	3.4	17707	43	1799	15
GO:0009888	tissue development	1.2E-05	1.0E-03	3.0	1.6	17707	544	1799	87
GO:0002576	platelet degranulation	1.2E-05	1.1E-03	3.0	2.4	17707	117	1799	28
GO:0045071	negative regulation of viral genome replication	1.3E-05	1.1E-03	3.0	3.0	17707	59	1799	18
GO:1903532	positive regulation of secretion by cell	1.4E-05	1.1E-03	3.0	1.9	17707	252	1799	48
GO:0030162	regulation of proteolysis	1.3E-05	1.1E-03	3.0	1.5	17707	729	1799	110
GO:0003008	system process	1.3E-05	1.1E-03	3.0	1.4	17707	1220	1799	169
GO:0030203	glycosaminoglycan metabolic process	1.4E-05	1.1E-03	2.9	2.2	17707	143	1799	32
GO:0071621	granulocyte chemotaxis	1.5E-05	1.2E-03	2.9	2.7	17707	76	1799	21
GO:0051336	regulation of hydrolase activity	1.5E-05	1.2E-03	2.9	1.4	17707	1231	1799	170
GO:0032330	regulation of chondrocyte differentiation	1.6E-05	1.3E-03	2.9	3.2	17707	49	1799	16
GO:0031401	positive regulation of protein modification process	1.6E-05	1.3E-03	2.9	1.4	17707	1156	1799	161
GO:0042325	regulation of phosphorylation	1.6E-05	1.3E-03	2.9	1.3	17707	1551	1799	207
GO:0022604	regulation of cell morphogenesis	1.6E-05	1.3E-03	2.9	1.6	17707	478	1799	78
GO:0002791	regulation of peptide secretion	1.7E-05	1.3E-03	2.9	1.8	17707	290	1799	53
GO:0030500	regulation of bone mineralization	1.7E-05	1.3E-03	2.9	2.8	17707	71	1799	20

GO:2000401	regulation of lymphocyte migration	1.7E-05	1.4E-03	2.9	3.0	17707	60	1799	18
GO:0051050	positive regulation of transport	1.8E-05	1.4E-03	2.9	1.4	17707	840	1799	123
GO:0051770	positive regulation of nitric-oxide synthase biosynthetic process	1.9E-05	1.5E-03	2.8	5.6	17707	14	1799	8
GO:0032675	regulation of interleukin-6 production	2.0E-05	1.5E-03	2.8	2.2	17707	139	1799	31
GO:0051707	response to other organism	2.0E-05	1.5E-03	2.8	1.6	17707	551	1799	87
GO:0017156	calcium ion regulated exocytosis	2.1E-05	1.6E-03	2.8	3.2	17707	50	1799	16
GO:0050708	regulation of protein secretion	2.1E-05	1.6E-03	2.8	1.8	17707	263	1799	49
GO:0098657	import into cell	2.2E-05	1.6E-03	2.8	1.5	17707	584	1799	91
GO:0007167	enzyme linked receptor protein signaling pathway	2.2E-05	1.6E-03	2.8	1.5	17707	664	1799	101
GO:0006023	aminoglycan biosynthetic process	2.3E-05	1.7E-03	2.8	2.4	17707	102	1799	25
GO:0008284	positive regulation of cell proliferation	2.3E-05	1.7E-03	2.8	1.4	17707	853	1799	124
GO:0002063	chondrocyte development	2.3E-05	1.7E-03	2.8	4.9	17707	18	1799	9
GO:0072676	lymphocyte migration	2.5E-05	1.8E-03	2.7	2.8	17707	67	1799	19
GO:0019935	cyclic-nucleotide-mediated signaling	2.6E-05	1.9E-03	2.7	2.1	17707	154	1799	33
GO:1903510	mucopolysaccharide metabolic process	2.8E-05	2.0E-03	2.7	2.4	17707	103	1799	25
GO:0006928	movement of cell or subcellular component	2.8E-05	2.1E-03	2.7	1.3	17707	1348	1799	182
GO:0050433	regulation of catecholamine secretion	2.9E-05	2.1E-03	2.7	3.2	17707	46	1799	15
GO:1901701	cellular response to oxygen-containing compound	3.0E-05	2.2E-03	2.7	1.4	17707	792	1799	116
GO:0032370	positive regulation of lipid transport	3.1E-05	2.2E-03	2.6	2.8	17707	68	1799	19
GO:0006820	anion transport	3.2E-05	2.3E-03	2.6	1.5	17707	542	1799	85
GO:0050806	positive regulation of synaptic transmission	3.4E-05	2.4E-03	2.6	2.2	17707	136	1799	30
GO:0048523	negative regulation of cellular process	3.3E-05	2.4E-03	2.6	1.2	17707	4609	1799	540
GO:0097530	granulocyte migration	3.5E-05	2.4E-03	2.6	2.5	17707	86	1799	22
GO:0061035	regulation of cartilage development	3.6E-05	2.5E-03	2.6	2.8	17707	63	1799	18
GO:1903531	negative regulation of secretion by cell	3.6E-05	2.5E-03	2.6	2.2	17707	130	1799	29
GO:0048566	embryonic digestive tract development	3.7E-05	2.6E-03	2.6	5.3	17707	15	1799	8
GO:0042339	keratan sulfate metabolic process	3.8E-05	2.6E-03	2.6	3.7	17707	32	1799	12
GO:0006897	endocytosis	3.9E-05	2.7E-03	2.6	1.6	17707	443	1799	72
GO:0050729	positive regulation of inflammatory response	3.9E-05	2.7E-03	2.6	2.2	17707	137	1799	30
GO:0045861	negative regulation of proteolysis	3.9E-05	2.7E-03	2.6	1.7	17707	321	1799	56
GO:0050801	ion homeostasis	4.0E-05	2.7E-03	2.6	1.5	17707	731	1799	108
GO:0050789	regulation of biological process	4.0E-05	2.7E-03	2.6	1.1	17707	10663	1799	1161
GO:0050795	regulation of behavior	4.1E-05	2.8E-03	2.6	2.9	17707	58	1799	17
GO:1905954	positive regulation of lipid localization	4.2E-05	2.8E-03	2.6	2.4	17707	93	1799	23
GO:0070555	response to interleukin-1	4.6E-05	3.1E-03	2.5	2.3	17707	106	1799	25
GO:0055065	metal ion homeostasis	4.8E-05	3.2E-03	2.5	1.5	17707	564	1799	87
GO:0045785	positive regulation of cell adhesion	4.8E-05	3.2E-03	2.5	1.6	17707	407	1799	67
GO:0051607	defense response to virus	4.8E-05	3.2E-03	2.5	2.0	17707	179	1799	36
GO:0071396	cellular response to lipid	5.1E-05	3.3E-03	2.5	1.6	17707	377	1799	63
GO:0006811	ion transport	5.2E-05	3.4E-03	2.5	1.3	17707	1207	1799	164

GO:0055080	cation homeostasis	5.2E-05	3.4E-03	2.5	1.5	17707	646	1799	97
GO:0050714	positive regulation of protein secretion	5.3E-05	3.4E-03	2.5	2.2	17707	126	1799	28
GO:0048247	lymphocyte chemotaxis	5.4E-05	3.5E-03	2.5	3.2	17707	43	1799	14
GO:0002793	positive regulation of peptide secretion	5.5E-05	3.5E-03	2.5	2.1	17707	146	1799	31
GO:0045667	regulation of osteoblast differentiation	5.7E-05	3.6E-03	2.4	2.2	17707	120	1799	27
GO:0098771	inorganic ion homeostasis	5.8E-05	3.7E-03	2.4	1.5	17707	656	1799	98
GO:0050926	regulation of positive chemotaxis	5.9E-05	3.7E-03	2.4	4.1	17707	24	1799	10
GO:0051480	regulation of cytosolic calcium ion concentration	6.0E-05	3.8E-03	2.4	1.7	17707	296	1799	52
GO:0097529	myeloid leukocyte migration	6.1E-05	3.8E-03	2.4	2.2	17707	114	1799	26
GO:0070167	regulation of biomineral tissue development	6.1E-05	3.8E-03	2.4	2.4	17707	89	1799	22
GO:2001023	regulation of response to drug	6.2E-05	3.9E-03	2.4	2.5	17707	83	1799	21
GO:0051048	negative regulation of secretion	6.2E-05	3.9E-03	2.4	2.1	17707	147	1799	31
GO:0043567	regulation of insulin-like growth factor receptor signaling pathway	6.7E-05	4.1E-03	2.4	4.4	17707	20	1799	9
GO:0042493	response to drug	6.7E-05	4.1E-03	2.4	1.5	17707	691	1799	102
GO:0035265	organ growth	7.1E-05	4.3E-03	2.4	3.7	17707	29	1799	11
GO:0044092	negative regulation of molecular function	7.2E-05	4.4E-03	2.4	1.4	17707	1069	1799	147
GO:0043086	negative regulation of catalytic activity	7.2E-05	4.4E-03	2.4	1.4	17707	750	1799	109
GO:1905521	regulation of macrophage migration*	7.6E-05	4.6E-03	2.3	3.3	17707	39	1799	13
GO:0007187	G protein-coupled receptor signaling pathway, coupled to cyclic nucleotide second messenger	7.7E-05	4.6E-03	2.3	1.9	17707	211	1799	40
GO:0098542	defense response to other organism	8.2E-05	4.9E-03	2.3	1.6	17707	360	1799	60
GO:0048871	multicellular organismal homeostasis	8.2E-05	4.9E-03	2.3	2.1	17707	129	1799	28
GO:0031589	cell-substrate adhesion	8.4E-05	5.0E-03	2.3	2.0	17707	156	1799	32
GO:0050922	negative regulation of chemotaxis	8.3E-05	5.0E-03	2.3	2.7	17707	61	1799	17
GO:0008016	regulation of heart contraction	8.4E-05	5.0E-03	2.3	1.8	17707	219	1799	41
GO:0002252	immune effector process	8.5E-05	5.0E-03	2.3	1.4	17707	878	1799	124
GO:0009615	response to virus	8.6E-05	5.0E-03	2.3	1.7	17707	285	1799	50
GO:0048646	anatomical structure formation involved in morphogenesis	8.9E-05	5.2E-03	2.3	1.5	17707	655	1799	97
GO:1901623	regulation of lymphocyte chemotaxis	8.9E-05	5.2E-03	2.3	3.9	17707	25	1799	10
GO:0002673	regulation of acute inflammatory response	9.0E-05	5.2E-03	2.3	2.4	17707	85	1799	21
GO:0050678	regulation of epithelial cell proliferation	9.1E-05	5.3E-03	2.3	1.7	17707	346	1799	58
GO:0055074	calcium ion homeostasis	9.2E-05	5.3E-03	2.3	1.6	17707	400	1799	65
GO:2000257	regulation of protein activation cascade	9.5E-05	5.5E-03	2.3	3.1	17707	45	1799	14
GO:0060337	type I interferon signaling pathway	9.7E-05	5.5E-03	2.3	2.8	17707	56	1799	16
GO:0099003	vesicle-mediated transport in synapse	9.9E-05	5.7E-03	2.2	2.3	17707	98	1799	23
GO:0010976	positive regulation of neuron projection development	1.0E-04	5.7E-03	2.2	1.7	17707	272	1799	48
GO:0016079	synaptic vesicle exocytosis	1.0E-04	5.8E-03	2.2	3.6	17707	30	1799	11
GO:0035296	regulation of tube diameter	1.0E-04	5.8E-03	2.2	2.2	17707	111	1799	25
GO:0035150	regulation of tube size	1.0E-04	5.8E-03	2.2	2.2	17707	111	1799	25
GO:0050794	regulation of cellular process	1.0E-04	5.8E-03	2.2	1.1	17707	9869	1799	1077

GO:0097746	regulation of blood vessel diameter	1.0E-04	5.8E-03	2.2	2.2	17707	111	1799	25
GO:1905048	regulation of metallopeptidase activity	1.1E-04	5.8E-03	2.2	4.2	17707	21	1799	9
GO:0050880	regulation of blood vessel size	1.0E-04	5.8E-03	2.2	2.2	17707	111	1799	25
GO:2001106	regulation of Rho guanyl-nucleotide exchange factor activity	1.1E-04	5.9E-03	2.2	9.8	17707	4	1799	4
GO:0010875	positive regulation of cholesterol efflux	1.1E-04	5.9E-03	2.2	4.2	17707	21	1799	9
GO:1904475	regulation of Ras GTPase binding	1.1E-04	5.9E-03	2.2	9.8	17707	4	1799	4
GO:2000741	positive regulation of mesenchymal stem cell differentiation	1.1E-04	5.9E-03	2.2	9.8	17707	4	1799	4
GO:0051952	regulation of amine transport	1.1E-04	6.0E-03	2.2	2.5	17707	80	1799	20
GO:1903034	regulation of response to wounding	1.1E-04	6.0E-03	2.2	1.9	17707	179	1799	35
GO:0035239	tube morphogenesis	1.1E-04	6.1E-03	2.2	1.8	17707	251	1799	45
GO:0090287	regulation of cellular response to growth factor stimulus	1.2E-04	6.3E-03	2.2	1.7	17707	281	1799	49
GO:1901615	organic hydroxy compound metabolic process	1.2E-04	6.5E-03	2.2	1.6	17707	435	1799	69
GO:0002237	response to molecule of bacterial origin	1.2E-04	6.6E-03	2.2	1.7	17707	289	1799	50
GO:0001822	kidney development	1.3E-04	6.6E-03	2.2	2.1	17707	132	1799	28
GO:0045860	positive regulation of protein kinase activity	1.3E-04	6.7E-03	2.2	1.5	17707	507	1799	78
GO:1902105	regulation of leukocyte differentiation	1.3E-04	6.7E-03	2.2	1.7	17707	267	1799	47
GO:0010812	negative regulation of cell-substrate adhesion	1.3E-04	6.8E-03	2.2	2.7	17707	63	1799	17
GO:0022409	positive regulation of cell-cell adhesion	1.3E-04	6.9E-03	2.2	1.7	17707	260	1799	46
GO:0001523	retinoid metabolic process	1.4E-04	7.2E-03	2.1	2.3	17707	100	1799	23
GO:1905606	regulation of presynapse assembly	1.4E-04	7.4E-03	2.1	3.5	17707	31	1799	11
GO:0099174	regulation of presynapse organization	1.4E-04	7.4E-03	2.1	3.5	17707	31	1799	11
GO:0007612	learning	1.6E-04	8.1E-03	2.1	2.1	17707	127	1799	27
GO:0009725	response to hormone	1.6E-04	8.2E-03	2.1	1.5	17707	616	1799	91
GO:0099601	regulation of neurotransmitter receptor activity	1.6E-04	8.3E-03	2.1	2.5	17707	76	1799	19
GO:0048714	positive regulation of oligodendrocyte differentiation*	1.6E-04	8.4E-03	2.1	4.0	17707	22	1799	9
GO:0003018	vascular process in circulatory system	1.7E-04	8.6E-03	2.1	2.0	17707	141	1799	29
GO:0006024	glycosaminoglycan biosynthetic process	1.7E-04	8.8E-03	2.1	2.3	17707	95	1799	22
GO:1902003	regulation of amyloid-beta formation	1.8E-04	9.0E-03	2.0	3.1	17707	42	1799	13
GO:0001817	regulation of cytokine production	1.8E-04	9.1E-03	2.0	1.4	17707	709	1799	102
GO:0050867	positive regulation of cell activation	1.8E-04	9.2E-03	2.0	1.6	17707	324	1799	54
GO:0010758	regulation of macrophage chemotaxis	1.9E-04	9.5E-03	2.0	3.7	17707	27	1799	10
GO:0051965	positive regulation of synapse assembly	1.9E-04	9.5E-03	2.0	2.7	17707	59	1799	16
GO:1903317	regulation of protein maturation	1.9E-04	9.5E-03	2.0	2.2	17707	102	1799	23
GO:0048660	regulation of smooth muscle cell proliferation	1.9E-04	9.5E-03	2.0	2.0	17707	142	1799	29
GO:0070244	negative regulation of thymocyte apoptotic process	1.9E-04	9.5E-03	2.0	7.0	17707	7	1799	5
GO:0007389	pattern specification process	2.0E-04	9.7E-03	2.0	1.6	17707	348	1799	57
GO:0050974	detection of mechanical stimulus involved in sensory perception	2.0E-04	9.7E-03	2.0	3.4	17707	32	1799	11

GO:0030593	neutrophil chemotaxis	2.0E-04	9.7E-03	2.0	2.5	17707	71	1799	18
GO:0007204	positive regulation of cytosolic calcium ion concentration	2.2E-04	1.1E-02	2.0	1.7	17707	258	1799	45
GO:0045859	regulation of protein kinase activity	2.2E-04	1.1E-02	2.0	1.4	17707	763	1799	108
GO:0060395	SMAD protein signal transduction	2.2E-04	1.1E-02	2.0	2.7	17707	54	1799	15
GO:0051247	positive regulation of protein metabolic process	2.3E-04	1.1E-02	2.0	1.3	17707	1594	1799	204
GO:0080134	regulation of response to stress	2.3E-04	1.1E-02	2.0	1.3	17707	1541	1799	198
GO:0045055	regulated exocytosis	2.3E-04	1.1E-02	2.0	1.4	17707	639	1799	93
GO:0035282	segmentation	2.3E-04	1.1E-02	2.0	3.0	17707	43	1799	13
GO:0032755	positive regulation of interleukin-6 production	2.3E-04	1.1E-02	2.0	2.4	17707	78	1799	19
GO:0061098	positive regulation of protein tyrosine kinase activity	2.4E-04	1.1E-02	2.0	2.6	17707	60	1799	16
GO:0014015	positive regulation of gliogenesis	2.4E-04	1.1E-02	2.0	2.5	17707	72	1799	18
GO:0030324	lung development	2.3E-04	1.1E-02	2.0	2.4	17707	78	1799	19
GO:0050863	regulation of T cell activation	2.4E-04	1.1E-02	2.0	1.6	17707	312	1799	52
GO:1902531	regulation of intracellular signal transduction	2.4E-04	1.1E-02	2.0	1.2	17707	1827	1799	230
GO:0050927	positive regulation of positive chemotaxis	2.5E-04	1.2E-02	1.9	3.9	17707	23	1799	9
GO:0010977	negative regulation of neuron projection development	2.5E-04	1.2E-02	1.9	2.0	17707	144	1799	29
GO:0019220	regulation of phosphate metabolic process	2.5E-04	1.2E-02	1.9	1.3	17707	1739	1799	220
GO:0051174	regulation of phosphorus metabolic process	2.5E-04	1.2E-02	1.9	1.3	17707	1739	1799	220
GO:0003014	renal system process	2.5E-04	1.2E-02	1.9	2.3	17707	91	1799	21
GO:0043901	negative regulation of multi-organism process	2.5E-04	1.2E-02	1.9	1.9	17707	158	1799	31
GO:0003006	developmental process involved in reproduction	2.5E-04	1.2E-02	1.9	1.5	17707	542	1799	81
GO:0006887	exocytosis	2.6E-04	1.2E-02	1.9	1.4	17707	716	1799	102
GO:0048638	regulation of developmental growth	2.7E-04	1.2E-02	1.9	1.6	17707	306	1799	51
GO:0019933	cAMP-mediated signaling	2.7E-04	1.3E-02	1.9	2.0	17707	138	1799	28
GO:1990266	neutrophil migration	2.8E-04	1.3E-02	1.9	2.4	17707	79	1799	19
GO:0043270	positive regulation of ion transport	2.8E-04	1.3E-02	1.9	1.7	17707	261	1799	45
GO:0030449	regulation of complement activation*	3.0E-04	1.3E-02	1.9	2.9	17707	44	1799	13
GO:0045778	positive regulation of ossification	3.0E-04	1.3E-02	1.9	2.3	17707	92	1799	21
GO:0007568	aging	3.0E-04	1.3E-02	1.9	1.8	17707	217	1799	39
GO:0090092	regulation of transmembrane receptor protein serine/threonine kinase signaling pathway	2.9E-04	1.3E-02	1.9	1.7	17707	239	1799	42
GO:0009628	response to abiotic stimulus	3.0E-04	1.3E-02	1.9	1.3	17707	1007	1799	136
GO:0032270	positive regulation of cellular protein metabolic process	2.9E-04	1.3E-02	1.9	1.3	17707	1522	1799	195
GO:0042135	neurotransmitter catabolic process	3.0E-04	1.3E-02	1.9	4.1	17707	19	1799	8
GO:0051769	regulation of nitric-oxide synthase biosynthetic process	3.0E-04	1.3E-02	1.9	4.1	17707	19	1799	8
GO:0071407	cellular response to organic cyclic compound	3.0E-04	1.3E-02	1.9	1.6	17707	346	1799	56
GO:0008347	glial cell migration*	3.0E-04	1.4E-02	1.9	4.1	17707	19	1799	8
GO:0010469	regulation of signaling receptor activity	3.1E-04	1.4E-02	1.9	1.9	17707	174	1799	33

GO:0001503	ossification	3.1E-04	1.4E-02	1.9	2.1	17707	112	1799	24
GO:1900272	negative regulation of long-term synaptic potentiation	3.2E-04	1.4E-02	1.9	5.4	17707	11	1799	6
GO:1903901	negative regulation of viral life cycle	3.3E-04	1.4E-02	1.8	2.3	17707	80	1799	19
GO:0036159	inner dynein arm assembly	3.4E-04	1.5E-02	1.8	4.6	17707	15	1799	7
GO:0016101	diterpenoid metabolic process	3.4E-04	1.5E-02	1.8	2.1	17707	106	1799	23
GO:0008037	cell recognition	3.4E-04	1.5E-02	1.8	2.0	17707	126	1799	26
GO:1901342	regulation of vasculature development	3.4E-04	1.5E-02	1.8	1.6	17707	379	1799	60
GO:0030154	cell differentiation	3.4E-04	1.5E-02	1.8	1.2	17707	1767	1799	222
GO:0071495	cellular response to endogenous stimulus	3.5E-04	1.5E-02	1.8	1.4	17707	680	1799	97
GO:1905523	positive regulation of macrophage migration	3.6E-04	1.5E-02	1.8	3.7	17707	24	1799	9
GO:0002920	regulation of humoral immune response*	3.5E-04	1.5E-02	1.8	2.5	17707	62	1799	16
GO:0050773	regulation of dendrite development	3.5E-04	1.5E-02	1.8	1.9	17707	147	1799	29
GO:0033344	cholesterol efflux	3.6E-04	1.5E-02	1.8	3.7	17707	24	1799	9
GO:0042551	neuron maturation	3.6E-04	1.5E-02	1.8	3.7	17707	24	1799	9
GO:0051668	localization within membrane	3.6E-04	1.5E-02	1.8	2.1	17707	113	1799	24
GO:0033280	response to vitamin D	3.6E-04	1.5E-02	1.8	3.7	17707	24	1799	9
GO:0032376	positive regulation of cholesterol transport	3.7E-04	1.6E-02	1.8	3.4	17707	29	1799	10
GO:0050866	negative regulation of cell activation	3.7E-04	1.6E-02	1.8	1.8	17707	190	1799	35
GO:0006874	cellular calcium ion homeostasis	3.7E-04	1.6E-02	1.8	1.6	17707	388	1799	61
GO:0032373	positive regulation of sterol transport	3.7E-04	1.6E-02	1.8	3.4	17707	29	1799	10
GO:0070613	regulation of protein processing	3.7E-04	1.6E-02	1.8	2.2	17707	100	1799	22
GO:0015711	organic anion transport	3.8E-04	1.6E-02	1.8	1.5	17707	420	1799	65
GO:0071675	regulation of mononuclear cell migration	3.8E-04	1.6E-02	1.8	2.8	17707	45	1799	13
GO:0060688	regulation of morphogenesis of a branching structure	3.8E-04	1.6E-02	1.8	2.8	17707	45	1799	13
GO:0032496	response to lipopolysaccharide	3.8E-04	1.6E-02	1.8	1.7	17707	272	1799	46
GO:0006898	receptor-mediated endocytosis	3.8E-04	1.6E-02	1.8	1.9	17707	176	1799	33
GO:0048519	negative regulation of biological process	3.9E-04	1.6E-02	1.8	1.1	17707	5258	1799	597
GO:1903035	negative regulation of response to wounding	3.9E-04	1.6E-02	1.8	2.3	17707	81	1799	19
GO:0030509	BMP signaling pathway	3.9E-04	1.6E-02	1.8	2.3	17707	81	1799	19
GO:1903555	regulation of tumor necrosis factor superfamily cytokine production	4.0E-04	1.6E-02	1.8	1.9	17707	148	1799	29
GO:0002697	regulation of immune effector process	4.2E-04	1.7E-02	1.8	1.6	17707	374	1799	59
GO:0045670	regulation of osteoclast differentiation	4.3E-04	1.7E-02	1.8	2.6	17707	57	1799	15
GO:1900449	regulation of glutamate receptor signaling pathway	4.3E-04	1.7E-02	1.8	2.5	17707	63	1799	16
GO:0008283	cell proliferation	4.3E-04	1.7E-02	1.8	1.5	17707	406	1799	63
GO:0034097	response to cytokine	4.3E-04	1.7E-02	1.8	1.5	17707	527	1799	78
GO:0002696	positive regulation of leukocyte activation	4.3E-04	1.7E-02	1.8	1.6	17707	312	1799	51
GO:0050796	regulation of insulin secretion	4.4E-04	1.8E-02	1.8	1.9	17707	163	1799	31
GO:0001763	morphogenesis of a branching structure	4.4E-04	1.8E-02	1.8	2.0	17707	135	1799	27
GO:0009410	response to xenobiotic stimulus	4.5E-04	1.8E-02	1.8	1.9	17707	142	1799	28

GO:2001236	regulation of extrinsic apoptotic signaling pathway	4.5E-04	1.8E-02	1.8	1.9	17707	149	1799	29
GO:0010769	regulation of cell morphogenesis involved in differentiation	4.5E-04	1.8E-02	1.8	1.6	17707	297	1799	49
GO:0009310	amine catabolic process	4.6E-04	1.8E-02	1.7	3.9	17707	20	1799	8
GO:0030850	prostate gland development	4.6E-04	1.8E-02	1.7	6.2	17707	8	1799	5
GO:0031620	regulation of fever generation	4.6E-04	1.8E-02	1.7	6.2	17707	8	1799	5
GO:0042402	cellular biogenic amine catabolic process	4.6E-04	1.8E-02	1.7	3.9	17707	20	1799	8
GO:0007160	cell-matrix adhesion	4.7E-04	1.8E-02	1.7	2.2	17707	95	1799	21
GO:0006875	cellular metal ion homeostasis	4.7E-04	1.8E-02	1.7	1.5	17707	504	1799	75
GO:0032940	secretion by cell	4.7E-04	1.8E-02	1.7	1.3	17707	872	1799	119
GO:0002021	response to dietary excess	4.6E-04	1.8E-02	1.7	6.2	17707	8	1799	5
GO:0010771	negative regulation of cell morphogenesis involved in differentiation	4.7E-04	1.8E-02	1.7	2.2	17707	95	1799	21
GO:0097009	energy homeostasis	4.8E-04	1.8E-02	1.7	3.1	17707	35	1799	11
GO:0045744	negative regulation of G protein-coupled receptor signaling pathway	4.8E-04	1.8E-02	1.7	3.1	17707	35	1799	11
GO:0009581	detection of external stimulus	4.9E-04	1.9E-02	1.7	2.0	17707	122	1799	25
GO:0071715	icosanoid transport	5.1E-04	1.9E-02	1.7	3.3	17707	30	1799	10
GO:1901571	fatty acid derivative transport	5.1E-04	1.9E-02	1.7	3.3	17707	30	1799	10
GO:0002009	morphogenesis of an epithelium	5.1E-04	1.9E-02	1.7	1.7	17707	268	1799	45
GO:0045604	regulation of epidermal cell differentiation	5.2E-04	2.0E-02	1.7	2.6	17707	58	1799	15
GO:0014075	response to amine	5.6E-04	2.1E-02	1.7	2.9	17707	41	1799	12
GO:0030574	collagen catabolic process	5.6E-04	2.1E-02	1.7	2.9	17707	41	1799	12
GO:0050918	positive chemotaxis	5.6E-04	2.1E-02	1.7	2.9	17707	41	1799	12
GO:0032680	regulation of tumor necrosis factor production	5.7E-04	2.1E-02	1.7	1.9	17707	144	1799	28
GO:0031650	regulation of heat generation	5.9E-04	2.2E-02	1.7	4.9	17707	12	1799	6
GO:1901654	response to ketone	5.8E-04	2.2E-02	1.7	1.8	17707	180	1799	33
GO:0030309	poly-N-acetyllactosamine metabolic process	5.9E-04	2.2E-02	1.7	4.9	17707	12	1799	6
GO:0016322	neuron remodeling*	5.9E-04	2.2E-02	1.7	4.9	17707	12	1799	6
GO:0040007	growth	6.2E-04	2.3E-02	1.6	1.6	17707	278	1799	46
GO:0001658	branching involved in ureteric bud morphogenesis	6.3E-04	2.3E-02	1.6	3.0	17707	36	1799	11
GO:2000241	regulation of reproductive process	6.3E-04	2.3E-02	1.6	2.0	17707	124	1799	25
GO:0003002	regionalization	6.2E-04	2.3E-02	1.6	1.7	17707	225	1799	39
GO:0022407	regulation of cell-cell adhesion	6.3E-04	2.3E-02	1.6	1.5	17707	412	1799	63
GO:0072507	divalent inorganic cation homeostasis	6.7E-04	2.5E-02	1.6	1.5	17707	437	1799	66
GO:0051043	regulation of membrane protein ectodomain proteolysis	6.8E-04	2.5E-02	1.6	3.8	17707	21	1799	8
GO:1902932	positive regulation of alcohol biosynthetic process	6.8E-04	2.5E-02	1.6	3.8	17707	21	1799	8
GO:0055082	cellular chemical homeostasis	6.9E-04	2.5E-02	1.6	1.4	17707	677	1799	95
GO:0072678	T cell migration	7.1E-04	2.6E-02	1.6	3.4	17707	26	1799	9

GO:0042417	dopamine metabolic process	7.1E-04	2.6E-02	1.6	3.4	17707	26	1799	9
GO:0061036	positive regulation of cartilage development	7.1E-04	2.6E-02	1.6	3.4	17707	26	1799	9
GO:0045687	positive regulation of glial cell differentiation	7.1E-04	2.6E-02	1.6	2.8	17707	42	1799	12
GO:1902991	regulation of amyloid precursor protein catabolic process	7.5E-04	2.7E-02	1.6	2.7	17707	48	1799	13
GO:1901215	negative regulation of neuron death	7.6E-04	2.7E-02	1.6	1.8	17707	190	1799	34
GO:0006721	terpenoid metabolic process	7.8E-04	2.8E-02	1.6	2.0	17707	112	1799	23
GO:0042476	odontogenesis	7.9E-04	2.8E-02	1.5	2.1	17707	92	1799	20
GO:0031349	positive regulation of defense response	8.0E-04	2.8E-02	1.5	1.5	17707	448	1799	67
GO:0031345	negative regulation of cell projection organization	8.1E-04	2.9E-02	1.5	1.8	17707	176	1799	32
GO:0090276	regulation of peptide hormone secretion	8.4E-04	3.0E-02	1.5	1.8	17707	191	1799	34
GO:0032332	positive regulation of chondrocyte differentiation	8.6E-04	3.0E-02	1.5	4.1	17707	17	1799	7
GO:0007178	transmembrane receptor protein serine/threonine kinase signaling pathway	8.5E-04	3.0E-02	1.5	1.8	17707	162	1799	30
GO:0017157	regulation of exocytosis	8.8E-04	3.1E-02	1.5	1.7	17707	199	1799	35
GO:0048839	inner ear development	8.9E-04	3.1E-02	1.5	2.8	17707	43	1799	12
GO:0043405	regulation of MAP kinase activity	9.0E-04	3.2E-02	1.5	1.6	17707	314	1799	50
GO:0042573	retinoic acid metabolic process	9.1E-04	3.2E-02	1.5	3.1	17707	32	1799	10
GO:0019722	calcium-mediated signaling	9.0E-04	3.2E-02	1.5	1.9	17707	134	1799	26
GO:0050982	detection of mechanical stimulus	9.3E-04	3.2E-02	1.5	2.6	17707	49	1799	13
GO:0071622	regulation of granulocyte chemotaxis	9.3E-04	3.2E-02	1.5	2.6	17707	49	1799	13
GO:0031623	receptor internalization	9.2E-04	3.2E-02	1.5	2.4	17707	61	1799	15
GO:0032642	regulation of chemokine production	9.3E-04	3.2E-02	1.5	2.2	17707	80	1799	18
GO:0045682	regulation of epidermis development	9.3E-04	3.2E-02	1.5	2.2	17707	80	1799	18
GO:0043068	positive regulation of programmed cell death	9.5E-04	3.3E-02	1.5	1.4	17707	633	1799	89
GO:0060736	prostate gland growth	9.6E-04	3.3E-02	1.5	5.5	17707	9	1799	5
GO:0036075	replacement ossification	9.7E-04	3.3E-02	1.5	3.3	17707	27	1799	9
GO:0030003	cellular cation homeostasis	9.6E-04	3.3E-02	1.5	1.4	17707	583	1799	83
GO:0001958	endochondral ossification	9.7E-04	3.3E-02	1.5	3.3	17707	27	1799	9
GO:0001659	temperature homeostasis	9.7E-04	3.3E-02	1.5	3.3	17707	27	1799	9
GO:0001774	microglial cell activation	9.7E-04	3.3E-02	1.5	3.3	17707	27	1799	9
GO:0002269	leukocyte activation involved in inflammatory response	9.7E-04	3.3E-02	1.5	3.3	17707	27	1799	9
GO:1905049	negative regulation of metallopeptidase activity	1.0E-03	3.4E-02	1.5	4.5	17707	13	1799	6
GO:0032941	secretion by tissue	1.0E-03	3.4E-02	1.5	4.5	17707	13	1799	6
GO:0070848	response to growth factor	1.0E-03	3.4E-02	1.5	1.6	17707	292	1799	47
GO:0045741	positive regulation of epidermal growth factor-activated receptor activity	1.0E-03	3.4E-02	1.5	4.5	17707	13	1799	6
