# Supplementary material for: Mesenchymal stem cell-neural progenitors are enriched in cell signaling molecules implicated in their therapeutic effect in multiple sclerosis
Source: PLoS One. 2023 Aug 11;18(8):e0290069. doi: 10.1371/journal.pone.0290069 (PMC10420335; doi:10.1371/journal.pone.0290069)
Supplement: S4 Table — (PDF) [file pone.0290069.s004.pdf]

Gene names – nervous system development (GO:0051960)				
ALK	DTX1	ITPKA	PC6	SHANK1
APOE	DUOXA1	JAM2	PHACTR1	SHH
APP	EDNRB	KALRN	PLAG1	SHOX2
ARHGAP44	EFHC2	KIAA0319	PLXNC1	SLC23A2
ATP1B2	EFNA1	KIF13B	PP1R9A	SLC6A4
AVIL	EFNB3	KNDC1	PPFIA2	SLITRK4
BAI1	EGR2	LINGO1	PPP3CA	SLITRK6
BAI3	EMA5B	LINGO2	PREX1	SNAP25
BHLHE41	EPHA4	LPHN1	PROX1	SNCA
BMP2	FAM150B	LPHN2	PTN	SORL1
BMP4	FAM19A3	LRFN5	PTPRD	SOX2
BMP5	FES	LRP1	PTPRZ1	SOX9
BRINP2	FEZ1	LRP4	RAB17	SPINT1
C16orf45	FGF20	LRRC4C	RARB	SPP1
CAMK1D	FGFR1	LRRK2	RASSF10	SRPX2
CAMK2B	FLRT2	LRRTM2	RELN	STX1B
CBLN2	FYN	MDGA1	RGMA	SYNDIG1
CCL3	FZD1	MIR146A	RGS2	SYT1
CLSTN2	FZD3	MYLIP	RIMS2	SYT17
CLSTN3	GAP43	NBL1	RND2	SYT3
CNR1	GNRH1	NEURL	ROR2	TBX6
COBL	GPER1	NLGN3	RTN4RL2	TENM4
COL3A1	GRHL3	NR2F1	SCARF1	THRB
CPNE5	GRIP1	NRCAM	SCN1B	TIMP2
CSF1	HECW1	NRG3	SEMA3A	TLR2
CSMD3	HES7	NTN1	SEMA3B	TNFRSF1B
CST7	HEY1	NTNG2	SEMA3D	TP73
CX3CL1	HEYL	NTRK1	SEMA6A	TSKU
CXCR4	HGF	NTRK2	SEMA6B	TYMP
DGKG	HOXA2	NUMB	SEMA6D	ZDHHC15
DLL1	IL1B	OMG	SERPINF1	ZHX2
DRAXIN	IRX3	PAX6	SERPINI1	ZNF365
DRD2	ISLR2	PBX1	SFRP2	ZSWIM5
