# Supplementary material for: Mesenchymal stem cell-neural progenitors are enriched in cell signaling molecules implicated in their therapeutic effect in multiple sclerosis
Source: PLoS One. 2023 Aug 11;18(8):e0290069. doi: 10.1371/journal.pone.0290069 (PMC10420335; doi:10.1371/journal.pone.0290069)
Supplement: S5 Table — (PDF) [file pone.0290069.s005.pdf]

Gene names – cell-cell signaling (GO:0007267)				
ADORA1	CNIH3	GABRR2	KCNMB4	PCSK1
AGT	CNR1	GAL3ST4	KIF5A	PCSK5
AMH	CX3CL1	GDF15	LILRB2	PGF
ANXA9	CXCL10	GJA3	LYNX1	PHEX
APOE	CXCL11	GJB2	MERTK	PMP22
AR	CXCL13	GNRH1	MPZ	PNOC
AREG	CXCL14	GPNMB	NAMPT	PTHLH
BMP2	DBH	GRID1	NLGN3	PTPRD
BMP3	DLG2	GRIK4	NLGN4X	RAPSN
BMP4	DLL1	GRIN2A	NMB	SEMA3B
CACNA1E	DRD1	GRM6	NOVA1	SFRP2
CBLN2	DRD2	GUCA1B	NPFF	SHH
CCL13	EFNA1	GUCY1A3	NPTX1	SLC12A6
CCL26	EFNB1	GUCY1B3	NPTX2	SLC12A7
CCL27	EFNB3	HGF	NTRK2	SLC17A7
CCL3	EGR3	HRH1	PCDHB10	SNAP25
CCL5	EREG	HRH2	PCDHB11	SNCA
CCL7	FGF10	HRH4	PCDHB13	SNCG
CCL8	FGF16	HTR7	PCDHB14	SYT1
CCR1	FGF18	IL1B	PCDHB16	SYT5
CD68	FGF20	IL7	PCDHB2	TNFAIP6
CHRM3	FGFBP2	KCNC4	PCDHB4	TNFSF10
CHRNA1	FZD1	KCND2	PCDHB5	TNFSF11
CHRNA9	GABRB2	KCNIP2	PCDHB6	TNFSF18
CLSTN3	GABRG3	KCNK3	PCDHB9	TP63
				WNT9A
