# Supplementary material for: Mesenchymal stem cell-neural progenitors are enriched in cell signaling molecules implicated in their therapeutic effect in multiple sclerosis
Source: PLoS One. 2023 Aug 11;18(8):e0290069. doi: 10.1371/journal.pone.0290069 (PMC10420335; doi:10.1371/journal.pone.0290069)
Supplement: S6 Table — (PDF) [file pone.0290069.s006.pdf]

Gene names – extracellular matrix organization (GO:0030198)				
A2M	COL3A1	IBSP	MFAP4	SH3PXD2B
ADAMTS19	COL6A2	ICAM1	MIA	SLC39A8
ADAMTS3	COL9A2	ICAM4	MMP11	SMOC1
ADAMTS4	COL9A3	ICAM5	MMP13	SMOC2
ADAMTS5	COLQ	ITGA1	MMP14	SMPD3
ADAMTS9	CSGALNACT1	ITGA10	MMP28	SOX9
ADAMTSL2	CTSK	ITGA2	MMP7	SPINT1
ADAMTSL3	CTSL	ITGA8	MYH11	SPOCK2
ADAMTSL4	DCN	ITGB7	NID1	SPP1
APP	FBLN1	JAM2	NPNT	SULF1
BCAN	FLRT2	LAMA2	NTN4	SULF2
COL13A1	FMOD	LAMA3	NTNG2	TGFB2
COL14A1	FOXC1	LAMA5	OLFML2A	TIMP1
COL15A1	FOXF1	LAMB3	PAPLN	TIMP2
COL18A1	GAS2	LTBP3	PECAM1	VCAM1
COL23A1	GPM6B	LUM	SERPINF2	VIT
COL24A1	HTRA1	MADCAM1	SFRP2	
