# Supplementary material for: Mesenchymal stem cell-neural progenitors are enriched in cell signaling molecules implicated in their therapeutic effect in multiple sclerosis
Source: PLoS One. 2023 Aug 11;18(8):e0290069. doi: 10.1371/journal.pone.0290069 (PMC10420335; doi:10.1371/journal.pone.0290069)
Supplement: Attachment — Submitted filename: AUTHOR RESPONSE LETTER.docx [file pone.0290069.s007.docx]

Author Response Letter

Journal Requirements:

1. Please ensure that your manuscript meets PLOS ONE's style requirements, including those for file naming. The PLOS ONE style templates can be found at 
https://journals.plos.org/plosone/s/file?id=wjVg/PLOSOne_formatting_sample_main_body.pdf and 
https://journals.plos.org/plosone/s/file?id=ba62/PLOSOne_formatting_sample_title_authors_affiliations.pdf

**Author response:** Confirmed.

2. We note that you have a patent relating to material pertinent to this article. Please provide an amended statement of Competing Interests to declare this patent (with details including name and number), along with any other relevant declarations relating to employment, consultancy, patents, products in development or modified products etc. Please confirm that this does not alter your adherence to all PLOS ONE policies on sharing data and materials, as detailed online in our guide for authors http://journals.plos.org/plosone/s/competing-interests by including the following statement: "This does not alter our adherence to  PLOS ONE policies on sharing data and materials.” If there are restrictions on sharing of data and/or materials, please state these. Please note that we cannot proceed with consideration of your article until this information has been declared.

This information should be included in your cover letter; we will change the online submission form on your behalf.

**Author response:** The declaration of competing interests has been included in the cover letter.

3. We note that you have included the phrase “data not shown” in your manuscript. Unfortunately, this does not meet our data sharing requirements. PLOS does not permit references to inaccessible data. We require that authors provide all relevant data within the paper, Supporting Information files, or in an acceptable, public repository. Please add a citation to support this phrase or upload the data that corresponds with these findings to a stable repository (such as Figshare or Dryad) and provide and URLs, DOIs, or accession numbers that may be used to access these data. Or, if the data are not a core part of the research being presented in your study, we ask that you remove the phrase that refers to these data.

**Author response:** Results section, page 10. The phrase “data not shown” was removed because there was no data to show. “*Gene ontology analysis did not reveal any significantly enriched pathways in any of the MSC groups ~~(data not shown)~~.*” In addition, the last sentence in this paragraph was removed entirely as it does not contribute to study conclusion: “*Other genes including Krüppel-like factor 15 (KLF15) and WD repeat domain 46 (WDR46) were detectable but not significantly different between the groups (data not shown)*.”

Results section, page 11. The phrase “data not shown” was removed because the data (number of differentially regulated genes) are presented in the text. The sentence is now consistent with the results narrative for Figure 1. “*In MSC-NPs from SPMS donors (MSCNP-SP) and from PPMS donors (MSCNP-PP), 295 DEGs and 67 DEGs were identified, respectively, compared to the MSCNP-C group ~~(data not shown)~~.”*

Results section, page 18. The phrase “data not shown” was removed because the gene names are presented in the text. “*Consistent with this finding, we observed that genes with the highest normalized read counts in both MSCs and MSC-NPs included ECM genes encoding collagen subtypes (COL1A1, COL1A2, COL3A1, COL4A1, COL4A2, COL5A1, COL6A1, COL6A2, COL6A3), fibronectin (FN1), fibrillin (FBN1), thrombospondin (THBS1, THBS2), galectin (LGALS1) and integrin (ITGA5, ITGB1), as well as genes encoding ECM remodeling enzymes including MMPs (MMP2, MMP14) and TIMPs (TIMP1, TIMP2, TIMP3) ~~(data not shown)~~*.”

Discussion section, page 21. At the suggestion of one of the reviewers, the following sentence was removed entirely from the revised manuscript because the point is sufficiently made without it. “*Furthermore, examination of the >100 MS donor-derived MSCs manufactured for autologous administration into clinical trial subjects (cliniclatrials.gov ID NCT01933802, NCT03355365 and NCT03822858) has not revealed any correlation between donor age or disease duration and population doubling time of MSCs (data not shown).*”

4. Please include your full ethics statement in the ‘Methods’ section of your manuscript file. In your statement, please include the full name of the IRB or ethics committee who approved or waived your study, as well as whether or not you obtained informed written or verbal consent. If consent was waived for your study, please include this information in your statement as well.

**Author response:** The following ethics statement has been included in the Methods section, page 5.

***“****Human bone marrow was obtained with ethics approval from Western IRB. Individuals who donated bone marrow provided written informed consent to participate in this study.”*

Reviewer #1: The use of MSC cells in regenerative medicine is a very important and promising field. The discovery of the mechanisms behind the positive effects is particularly important when the cell products are put into clinical trials. From this point of view, the work can provide useful and interesting results in the exploration of the mechanism of MSCs and MSC-NPs. Although the number of samples is low in this study (as the authors properly point it out),
I consider it important that the samples came from the already completed clinical trial, so no further invasive intervention was necessary.
The data seem to be well processed, and the conclusions are moderate.
I would like to make only a few comments, mainly concerning the discussion part as follows:
- in Figure 1A, although I agree that there is no difference between the MS and control groups, it is not clear why the authors say that the samples are clustered into one group? Rather, it cannot be interpreted as clustering or interpret what it means in this case if they are not clustered together.

**Author response:** The reviewer is correct in pointing out that the term ‘clustering’ was inappropriately used in the sentence. The Results section on page 10 of the manuscript was revised to say the following “*PCA analysis (Fig 1) showed minimal variance between MSCs across groups*.”

The differences between MS donors are addressed again in the discussion "Furthermore, examination of the >100 MS donor-derived MSCs manufactured for autologous administration into clinical trial subjects (cliniclatrials.gov ID NCT01933802, NCT03355365 and NCT03822858) has not revealed any correlation between donor age or disease duration and population doubling time of MSCs (data not shown).” That's a very strong claim without presenting the data, especially as this is a controversial issue in the field. I recommend rephrasing the sentence or providing supporting data.

**Author response:** The above-mentioned sentence has been removed from the revised manuscript, as the point is sufficiently made without it.

- Due to the spheroid shape, it is completely understandable that the expression of extracellular matrix genes changes in MSC-NPs, but what relevance can this have in vivo conditions? This should also be addressed in the discussion.

**Author response:** The following paragraph and references were added to the Discussion page 22:

*“ECM proteins are abundantly expressed in MSCs where they are well-known to play a key role in MSC-mediated tissue regeneration by restoring tissue structure, directing cell migration, promoting angiogenesis and stimulating nerve growth [14]. Although the upregulation of ECM proteins in MSC-NPs is likely related to their spheroid morphology, there is evidence that they also play a role in enhanced cell survival and regenerative function [22]. TIMP-1, for example, has been shown to promote oligodendrocyte differentiation and enhance CNS myelination, suggesting that MSC-NP-derived TIMP-1 may play a therapeutic role in the context of MS [23, 24]. Similarly, osteopontin, which is significantly upregulated in MSC-NPs, has been shown to promote oligodendrocyte proliferation and differentiation suggesting that it is beneficial to remyelination [25]. While further validation is required, the upregulation of ECM proteins in MSC-NPs points to a potential therapeutic mechanism by which MSC-NPs may modify neuroinflammation and remyelination in MS.”*

Reviewer #2: The paper was described correctly and clearly. Unfortunately, with 2 control donors, it is difficult to draw conclusions. However, the main experimental gaps relate to further research. The evaluation of gene expression seems to be only the beginning. Subsequently, the level of proteins and the secretory properties of the cells should be assessed.
Changes in gene expression in differentiated cells are obvious. The question is whether they have an impact on the secretion and potential therapeutic properties of the cells.
If the authors carry out further experiments, then the paper will be very interesting.

**Author response:** We have confirmed increased protein expression/secretion for several of the genes described in manuscript, including PEDF and HGF in Figure 4, and TIMP-1 and Osteopontin in Figure 6. Additional validation of protein secretion and the contribution of these proteins to the therapeutic properties of MSC-NPs, while outside the scope of this particular study, is an important future direction of this research. We revised the last sentence in the manuscript discussion to highlight this point. *“Whether or not these gene expression differences translate into increased secretion of specific proteins that impact the potency or therapeutic efficacy of MSC-NP-based cell therapy remains to be determined.”*
